# Long noncoding RNA LINC01606 protects colon cancer cells from ferroptotic cell death and promotes stemness by SCD1–Wnt/β‐catenin–TFE3 feedback loop signalling

**DOI:** 10.1002/ctm2.752

**Published:** 2022-04-29

**Authors:** Yajun Luo, Siqi Huang, Jinlai Wei, He Zhou, Wuyi Wang, Jianguo Yang, Qican Deng, Hao Wang, Zhongxue Fu

**Affiliations:** ^1^ Department of Gastrointestinal Surgery The First Affiliated Hospital of Chongqing Medical University Chongqing China; ^2^ Department of Gastrointestinal Surgery The Affiliated Hospital of North Sichuan Medical College Nanchong Sichuan China; ^3^ Department of Gastrointestinal Surgery Sichuan Cancer Hospital and Institute Chengdu Sichuan China

**Keywords:** colon cancer, ferroptosis, LINC01606, lipid peroxidation, SCD1, Wnt/β‐catenin

## Abstract

**Background:**

Ferroptosis is principally caused by iron catalytic activity and intracellular lipid peroxidation. Long noncoding RNAs (lncRNAs) play crucial roles in tumorigenesis. However, the potential interplay between lncRNA LINC01606 and ferroptosis in colon cancer remains elusive.

**Methods:**

The expression level of LNC01606 in colon cancer tissue was detected by quantitative real‐time polymerase chain reaction. The functional role of LNC01606 was investigated by gain‐ and loss‐of‐function assays both in vitro and in vivo. The LINC01606‐SCD1‐Wnt/β‐catenin‐TFE3 axis were screened and validated by DNA/RNA pull down, gas chromatography‐mass spectrometry, RNA immunoprecipitation and dual‐luciferase reporter.

**Results:**

The expression of lncRNA LINC01606 was frequently upregulated in human colon cancer and strongly associated with a poor prognosis. LINC01606 functioned as an oncogene and promotes colon cancer cell growth, invasion and stemness both in vitro and in vivo. Moreover, LINC01606 protected colon cancer cells from ferroptosis by decreasing the concentration of iron, lipid reactive oxygen species, mitochondrial superoxide and increasing mitochondrial membrane potential. Mechanistically, LINC01606 enhanced the expression of stearoyl‐CoA desaturase 1 (SCD1), serving as a competing endogenous RNA to modulate miR‐423‐5p expression, subsequently activating the canonical Wnt/β‐catenin signaling, and transcription factor binding to IGHM enhancer 3 (TFE3) increased LINC01606 transcription after recruitment to the promoter regions of LINC01606. Furthermore, we confirmed that upregulated LINC01606 and Wnt/β‐catenin formed a positive feedback regulatory loop, further inhibiting ferroptosis and enhancing stemness.

**Conclusions:**

LINC01606 functions as an oncogene to facilitate tumor cell stemness, proliferation and inhibit ferroptosis and is a promising therapeutic target for colon cancer.

## Graphical abstract

1. LINC01606 functions as an oncogene to facilitate malignant phenotypes of colon cancer cells by enhancing tumour cell stemness and anti‐ferroptosis by Wnt/β‐catenin signalling;
2. LINC01606 protects colon cancer cells from ferroptotic cell death by increasing the formation of monounsaturated fatty acids;
3. LINC01606–Wnt/β‐catenin–TFE3 positive feedback regulatory loop signalling blocks ferroptosis and promotes stemness.

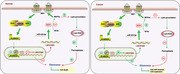

## 1 INTRODUCTION

Ferroptosis, a recently defined type of regulated cell death (RCD), is iron dependent and is featured via the accumulation of lethal intracellular lipid reactive oxygen species (ROS).^1^ It differs from other types of cell death, such as autophagy, necroptosis and various other forms of RCD, in morphology, genetics and biochemistry.^2^, ^3^ Cellular metabolism plays a central role in ferroptosis: the role of increasing iron accumulation and lipid peroxidation as the executioners of ferroptosis, in contrast to the role of the cystine‐import GSH–GPX4 axis in suppressing ferroptosis.^4^, ^5^ During ferroptosis, toxic lipid ROS accumulate from the reaction between iron catalytic activity and lipid peroxidation, which are themselves generated via the oxidation of polyunsaturated fatty acids (PUFAs) in mitochondria, endoplasmic reticulum and lysosomes.^6^, ^7^ A large number of human tumours have been related to the abnormal function of ferroptotic cell death, including colon cancer.^8^ ^–11^ These studies have highlighted the function of ferroptotic cell death in tumour suppression and resistance to chemo/radio/immunotherapy.^2^, ^8^ Notably, the relationship between ferroptosis and cancer stem cells (CSCs) has been identified in colon cancer. For instance, targeting SLC7A11 attenuates the chemoresistance and stemness of colon cancer cells through triggering ferroptotic cell death,^12^ dichloroacetate attenuates the stemness of colon cancer cells^13^ and Betulaceae extract effectively inhibits colon cancer cells by triggering ferroptosis.^14^ CSCs are a small subgroup of tumour cells responsible for cancer initiation, drug resistance and recurrence.^15^ Therefore, revealing the underlying molecular mechanism of the ferroptosis and stemness process involved in colon cancer is a compelling need for the development of more effective therapeutic strategies against this cancer and improvement of the overall prognosis.

Cancer cells exhibit a distinct metabolic phenotype to support survival and growth. The metabolic reprogramming of cancer cells provides sufficient energy, phospholipids, amino acids and other macromolecules to meet the needs for fast proliferation, continuous growth and survival under suboptimal conditions that provide insufficient nutrition within the tumour microenvironment.^16^ In recent years, changed lipid metabolism in tumour cells has gradually attracted increasing attention. In contrast to normal cells, which heavily tend to take up fatty acids from exogenous sources, tumour cells prefer to depend mainly on de novo lipogenesis.^17^ When lipid synthesis is increased in cancer cells, more lipid biosynthesis enzymes are also needed to generate diverse fatty acids (FAs). Stearoyl‐CoA desaturase 1 (SCD1) is the crucial enzyme in de novo FA synthesis and catalyses the desaturation of saturated fatty acids (SFAs) to monounsaturated fatty acids (MUFAs), principally palmitic acid and stearic acid, to their monounsaturated counterparts, palmitoleic acid and oleic acid, respectively.^18^, ^19^ Extensive studies have shown the influence of SCD1 on ferroptosis and survival in CSCs, which is tightly related to the FA balance in ovarian cancer,^18^, ^19^ gastric cancer,^20^ pancreatic cancer,^21^ hepatocellular cancer^22^ and lung cancer.^23^ Furthermore, SCD1 increases the production of lipid‐modified Wnt proteins by MUFAs that activate the Wnt signalling, which is essential in stem cells.^24^ Therefore, targeting SCD1 appears to be a promising strategy to overcome cancer progression by inducing ferroptosis. However, the effects of targeting SCD1 to reverse cancer progression by inducing ferroptosis have not been well clarified in colon cancer.

Notably, long noncoding RNAs (lncRNAs) are no coding potential transcripts longer than 200 nucleotides,^25^ and multiple lines of evidence have recently highlighted the role of lncRNAs in tumour hallmarks, such as migration, apoptosis, stemness, drug resistance and particularly ferroptosis.^3^, ^26^, ^27^ For instance, lncRNA LINC00336 acts as an oncogene to inhibit ferroptosis and promote cell proliferation,^28^ lncRNA MT1DP functions as a tumour suppressor to sensitise ferroptosis in lung cancer cells,^29^ and lncRNA LINC00618 accelerates ferroptosis in human leukaemia cells.^30^ In a previous study, we confirmed that lncRNA LINC01606 promotes gastric cancer progression via activating Wnt/β‐catenin signalling.^31^ However, the molecular mechanisms involving LINC01606 in Wnt/β‐catenin signalling and ferroptosis are not clear. In line with the above notion, we hypothesised that LINC01606 could protect colon cancer cells from ferroptotic cell death and promote stemness via SCD1–Wnt/β‐catenin signalling. Here, we present evidence that LINC01606 was upregulated in colon cancer and predicted a poor prognosis. Furthermore, LINC01606 was found to interact with miR‐423‐5p to contribute to stemness and to suppress the antitumour effect of ferroptosis inducers by modulating SCD1 expression, as a result reprogramming lipid metabolism and activating Wnt/β‐catenin signalling through increasing cellular MUFAs to maintain the intracellular redox balance. In turn, activation of Wnt/β‐catenin signalling enhanced LINC01606 expression by directly binding to the transcription factor IGHM enhancer 3 (TFE3), which formed a positive feedback loop. Here, this study will provide important theoretical evidence for explaining the mechanisms of the LINC01606–SCD1–Wnt/β‐catenin axis in colon cancer progression and simultaneously provide a new biomarker and target for cancer therapy.

## 2 METHODS

### 2.1 Patients and tumour tissues

A total of 83 colon cancer patients were enrolled in this study. All tumour tissues and paired adjacent noncancerous tissues were obtained at the time of surgery in the First Affiliated Hospital of Chongqing University (Chongqing, China) from September 2019 and September 2020. The detailed clinicopathologic parameters of these patients were collected and informed consent was obtained from all patients. The study protocol was approved by the Ethics Committee of the First Affiliated Hospital of Chongqing University, Chongqing, China.

### 2.2 Cell lines, cell cultures and chemicals

Human colon cancer cell lines SW480 and HT29, and human HEK293T cells were purchased from the Chinese Academy of Cell Bank (Shanghai, China). Cells were cultured in Dulbecco's modified Eagle's medium (DMEM; Gibco, Gaithersburg, MD, USA) supplemented with 10% foetal bovine serum (FBS; Gibco) and 100 U/ml penicillin and 100 μg/ml streptomycin (Gibco) at 37°C in an incubator with a humidified atmosphere with 5% CO_2_. All cell lines were authenticated before use by short tandem repeat profiling. Ferrostatin‐1 (Fer‐1), Erastin, RSL3, C59, BML‐284 and 5‐Fluorouracil (5‐Fu) were purchased from MedChemExpress (MCE, Monmouth Junction, NJ, USA).

### 2.3 Lentivirus, plasmid transfection and RNA interference

To suppress LINC01606, lentiviral‐mediated short hairpin RNA (Lv‐shRNA) clones targeting LINC01606 and shRNA non‐silencing control were synthesised by Genechem (Shanghai, China). To overexpress LINC01606, the full‐length cDNA of LINC01606 and empty vector were synthesised and cloned into GV367 vector (Genechem). Stable expressing clones were selected using 4 μg/ml puromycin. The pcDNA3‐TFE3 plasmids, the wild‐type (WT) and mutant variants of LINC01606 plasmids, the WT and mutant variants of LINC01606 promoter plasmids and the 3′UTR of the WT and mutant variants SCD1 in miR‐423‐5p binding sites were cloned into psi‐Check2 vector by Hanbio Biotechnology (Shanghai, China). SuperTOPFlash plasmid, SuperFOPFlash plasmid and pRL‐TK plasmid were purchased from Beyotime Biotechnology (Shanghai, China). miR‐423 mimics, mimics negative control (NC), miR‐423 inhibitors and inhibitors NC were purchased from Sangon Biotech (Shanghai, China). siRNA against LEF1, TCF7 (Ruibio, Guangzhou, China) and a non‐targeting siRNA control (Ruibio) were used to knock down gene expression. Plasmid transfections and the oligonucleotides were performed according to the manufacturer's instructions for Lipofectamine 2000 (Invitrogen, Carlsbad, CA, USA). Sequences for shRNAs or siRNA involved were listed in Table S1.

### 2.4 Quantitative real‐time polymerase chain reaction

Total RNA was extracted using TRIzol reagent (Invitrogen) and assessed using NanoDrop 2000 Spectrophotometer (Thermo Fisher, Waltham, MA, USA), and RNA was reversed transcribed using the Transcriptor First Strand cDNA Synthesis Kit (Roche, Basel, Switzerland) according to the respective manufacturer's instructions. Quantitative polymerase chain reaction (PCR) was conducted using the standard SYBR Green master mix (Roche) according to the manufacturer's protocol. GAPDH and U6 were used as a reference gene for mRNA and miRNA, respectively. The primer sequences used in the study were listed in Table S2.

### 2.5 Cell viability and colony formation assays

The cell viability assay was performed by Cell Counting Kit‐8 (CCK‐8; Beyotime Institute of Biotechnology, Shanghai, China) according to the manufacturer's protocol. Briefly, cells were seeded in 96‐well plates each well at a density of 5 × 10^3^ cells/well in triplicate, and the absorbance was measured on a microplate reader (Bio‐Rad, Hercules, CA, USA) at 450 nm of each well. For the cell colony formation assay, 200 cells were seeded in 12‐well plates and cultured in medium. After 2 weeks, cells were fixed with 4% paraformaldehyde (Sigma–Aldrich, St. Louis, MO, USA) and stained with 1% crystal violet (Beyotime Institute of Biotechnology). The number of visible cell colonies was counted from three dishes.

### 2.6 Transwell and apoptosis assays

Cell migration assays were performed using a 24‐well transwell chamber and separated by polycarbonate membranes with 8 μm pores (Corning, Tewksbury, MA, USA). Then, cells were seeded in the upper chamber at a density of 5 × 10^3^, and the lower chamber was filled with culture medium containing 10% FBS. Migrating cells attached to the lower membrane surface were fixed with 4% paraformaldehyde and stained with 1% crystal violet after incubated for 48 h. Cell invasion assays were conducted in a similar fashion with Matrigel (BD Pharmingen, San Jose, CA, USA) coating. Three random fields were counted per experiment. Cell apoptosis assays were assayed by flow cytometry using the Annexin V‐APC and 4′,6‐diamidino‐2‐phenylindole (DAPI; BD Biosciences, San Jose, CA, USA) based on the manufacturer's procedures. The apoptotic cells were analysed on a BD Beckman cytometer (Beckman Coulter Life Sciences, Miami, FL, USA) and FlowJo software.

### 2.7 Sphere formation assay

Single‐cell suspensions were seeded into six‐well low‐attachment plates (Corning) and cultured in serum‐free medium DMEM/F12 (Gibco) supplemented with 2% B27 (Invitrogen), 20 ng/ml human recombinant epidermal growth factor (EGF; Sigma–Aldrich) and 10 ng/ml basic fibroblast growth factor (bFGF; Sigma–Aldrich) for 14 days. The spheroids were photographed and counted under Olympus microscope (Olympus Corporation, Tokyo, Japan).

### 2.8 Flow cytometry

Fluorescence‐activated cell sorting (FACS) was performed using standard protocols. Briefly, cells were stained with human anti‐CD44–APC antibody (Biolegend, San Diego, CA, USA) and human anti‐CD133‐FITC antibody (Biolegend) at 4°C in the dark for 15 min. Separate aliquots of cells were single labelled with human anti‐CD44–APC antibody and human anti‐CD133–FITC used as controls. Then, the cells were washed by PBS three times and resuspended in PBS with 2% FBS. The cell suspensions were applied onto a BD Facs Aria IIIu Flow Cytometer (BD Bioscience Franklin Lakes, NJ, USA).

### 2.9 Xenograft model

Four‐week‐old female nude mice were purchased from Cavens (Changzhou, China). Nude mice were randomly divided into four groups. SW480 and HT29 cells infected with sh‐LINC01606, Lv‐LINC01606 and control vectors were subcutaneously implanted in mice (*n* = 6 per group), respectively. Tumour volumes (*V* = length × width^2^/2) were measured every 3 days, and tumour weights were determined after 4 weeks. Studies on all animal's experimental procedures were conducted with approval from the Animal Research Ethics Committee of Chongqing Medical University.

### 2.10 Transmission electron microscopy

Cells were collected and immediately fixed electron microscopy fixative solution containing 2.5% glutaraldehyde for 2 h at 4°C. The fixed cells were pre‐embedded in 1% agarose solution, then post‐fixed with 1% osmium tetroxide at room temperature for 2 h. After dehydration through a graded ethanol series, the cells were embedded in EMBed 812 for 5–8 h. Then, the samples were inserted into the embedding plate for 37°C overnight. The resin blocks were cut into 60–80 nm ultra‐thin slices on the ultra‐thin microtome. After 2% uranium acetate saturated alcohol solution avoided light staining for 8 min and 2.6% lead citrate avoided CO_2_ staining for 8 min, the cuprum grids were put into the grids board and dried overnight at room temperature. Images were acquired using transmission electron microscope (Hitachi, Taitou‐ku, Tokyo, Japan).

### 2.11 Iron measurements

The ferrous irons (Fe^2+^) or total irons were measured by Iron Assay Kit (Sigma–Aldrich) according to the manufacturer's instructions. Briefly, cells (2 × 10^6^) were rapidly homogenised in 4–10 volumes of iron assay buffer and removed insoluble material by centrifuging at 16 000×*g* for 10 min at 4°C. To measure Fe^2+^, 50 μl samples was added to sample wells in a 96‐well plate and the volume was brought to 100 μl per well with iron assay buffer, and 5 μl of iron assay buffer was added to each sample. To measure total iron, 50 μl samples was added in a 96‐well plate and the total volume was brought to 100 μl per well with iron assay buffer, and 5 μl of iron reducer was added to each of the sample wells to reduce Fe^3+^ to Fe^2+^. Then, samples were incubated for 30 min at 25°C in dark conditions. After adding 100 μl of iron probe to each well, samples were mixed well by pipetting and incubated for 1 h at room temperature in dark conditions. Finally, the absorbance was detected at 593 nm (A593).

### 2.12 Lipid ROS measurements

Cells were treated as indicated, and 50 μM C11‐BODIPY Lipid Peroxidation Sensor (Thermo Fisher; Cat #D3861) was added and incubated for 1 h and protected from light. Excess C11‐BODIPY was removed from cells by washing the cells twice with PBS, and labelled cells were trypsinised, resuspended in PBS with 5% FBS. The fluorescence of C11‐BODIPY581/591 was analysed using a flow cytometer.

### 2.13 Mitochondrial membrane potential measurements

The mitochondrial membrane potential was measured by using Mitochondrial Membrane Potential Assay Kit (Beyotime Institute of Biotechnology). Cells were treated as indicated, then 50 μl of JC‐1 dye in 2 ml staining buffer and 8 ml ultrapure water were added to the cells and incubated for 20 min at 37°C in dark conditions. Cells were washed twice with the staining buffer to remove excess JC‐1 and resuspended in the staining buffer. Then, samples were added in black 96‐well plates and read on a microplate reader. The fluorescence intensity levels (Green: *λ*_ex_ = 490/*λ*_em_ = 530 nm) and (Red: *λ*_ex_ = 525/*λ*_em_ = 590 nm) were monitored for ratio analysis. The ratio of Red/Green fluorescence intensity was used to determine mitochondrial membrane potential.

### 2.14 Mitochondrial superoxides measurements

Mitochondrial superoxides‐targeted MitoSOX™ Red mitochondrial superoxide indicator for live‐cell imaging (Invitrogen) was used to measure mitochondrial superoxides accumulation according to the manufacturer's protocol. Briefly, cells were grown on glass cover slips in a six‐well plate and 1 ml of 5 μM MitoSOX™ reagent working solution was added to cover cells adhering to cover slips after indicated treatment. Cells were incubated for 10 min at 37°C and protected from light, then cells were washed gently three times with the warm buffer. Cover slips were mounted in the warm buffer for imaging using confocal microscope (Leica, Wetzlar, Germany).

### 2.15 Fluorescence in situ hybridisation

Cy3‐labelled LINC01606 probes were synthesised and fluorescent in situ hybridisation (FISH) Kit was purchased in RiboBio (Guangzhou, China). Briefly, cells were grown on glass cover slips and fixed with 4% paraformaldehyde and hybridised overnight with the probe. Stain cell nuclei were stained with 1 μg/ml DAPI. Images were acquired on a confocal fluorescence microscope (Leica, Wetzlar, Germany).

### 2.16 Dual‐Luciferase reporter assays and TopFlash/FopFlash reporter assays

The WT and mutant variants LINC01606 or the 3′‐UTR of the WT and mutant variants SCD1 plasmids were cotransfected concurrently with miR‐423‐5p mimics and NCs after seeded into six‐well plates for 24 h. Luciferase assays were performed using a Dual‐Luciferase reporter assay kit (Beyotime Biotechnology) as described in the protocols of manufacturer after transfection for 48 h. The luciferase activities were measured on the luminometer microplate reader (Thermo Fisher) following the manufacturer's instructions.

For the TOPFlash/FOPFlash reporter assay, cells were cultured in six‐well plate for 24 h and cotransfected with 1 μg of SuperTOPFlash plasmid, SuperFOPFlash plasmid using Lipofectamine 2000 (Invitrogen) according to the manufacturer's instructions, respectively. Meanwhile, 0.5 μg pRL‐TK Renilla control luciferase plasmid was cotransfected to normalise for transfection efficiency. After transfection for 48 h, cells were lysed using lysis buffer, and luciferase activity was assayed in a luminometer using the Dual‐Luciferase reporter assay kit (Beyotime Biotechnology). Topflash and Fopflash luciferase values were normalised to Renilla luciferase activity, and values were normalised to control.

### 2.17 Western blot

Cells were collected and lysed in RIPA buffer (Beyotime) on ice. Total proteins were quantified with the BCA assay kit (Beyotime). Samples were separated using sodium dodecyl sulphate‐polyacrylamide gels and then transferred to polyvinylidene fluoride membrane (Merck Millipore, Darmstadt, Germany) by standard procedures. The membranes were incubated with the relevant primary antibody overnight at 4°C. The protein labels were detected by enhanced chemiluminescence (ECL) solution (Beyotime). The primary antibodies included SCD1 (1:1000; Abcam, Cambridge, MA, USA), Wnt3a (1:2000; Abcam), β‐catenin (1:1000; Abcam), c‐Myc (1:1000; Proteintech, Wuhan, China), TCF7 (1:500; Proteintech), LEF1 (1:1000; Proteintech), EP300 (1:500; Santa Cruz, CA, USA) and GAPDH (1:1000; Abcam).

### 2.18 RNA pull‐down assay

Biotin‐labelled probes for WT‐bio‐miR‐423‐5p and mutant (MUT)‐bio‐miR‐423‐5p, and corresponding NC‐bio‐probe were designed and synthesised by Sangon Biotech. Cells were further transfected with 50 nM biotin‐labelled NC‐bio‐probe, WT‐bio‐miR‐423‐5p and MUT‐bio‐423‐5p for 48 h, respectively. Then, cells were harvested and incubated in specific lysis buffer (Ambion, Austin, Texas, USA) for 10 min. The lysate was incubated with M‐280 streptavidin magnetic beads (S3762; Sigma–Aldrich) at 4°C overnight. And then the beads were washed twice with precooled lysis buffer, three times with low salt buffer and once with high salt buffer. The binding RNA was extracted by Trizol, and the enrichment of LINC01606 and SCD1 was detected by RT‐qPCR.

### 2.19 RNA immunoprecipitation (RIP) assay

The binding between LINC01606 or miR‐423‐5p and Argonaute‐2 (Ago2) was detected using the EZ‐Magna RIP™ RNA‐Binding Protein Immunoprecipitation Kit (Merck Millipore, Billerica, MA, USA) according to the manufacturer's instructions. Cells were lysed with RIP lysis buffer containing protease inhibitor cocktail, and then the cell supernatants were incubated with anti‐Ago2 (ab186733; Abcam) antibody or anti‐immunoglobulin G antibody (ab182931; Abcam) conjugated magnetic beads overnight at 4°C. Then, the beads were washed six times with cold RIP Wash Buffer, and proteinase K buffer was used to remove protein in the RIP complex. Phenol:chloroform:isoamyl alcohol were consecutively used to extract RNA from magnetic beads. The enrichment of LINC01606, SCD1 and miR‐432‐5p were detected by qRT‐PCR.

### 2.20 Gas chromatography–mass spectrometry‐based lipidomics

Cells were scraped off the plate and frozen in liquid nitrogen, and then were subjected to lipid extraction. Lipids were extracted using 1 ml of chloroform methanol solution for 30 min ultrasound, then 1% sulphuric acid–methanol solution 2 ml for 30 min methyl esterification at 4°C and 1 ml of N‐hexane for extraction. The 500 μl supernatant of the extract was mixed with 25 μl of internal standard (methyl nonadecanoate) before injection. Gas chromatography–mass spectrometry (GC–MS) analysis was carried out using a Agilent 7890/5975C GC–MS system (Agilent Technologies, Santa Clara, CA, USA) for single ion monitoring‐based analysis. Four independent biological replicates were performed for each condition. Five hundred microliters of supernatant of the extract was mixed with 25 μl of internal standard (methyl nonadecanoate) before injection.

### 2.21 Promoter DNA pull‐down and MS

The promoter region of LINC01606 was amplified by PCR using 5′‐biotin‐TEG‐labelled primer. The last exon and intron region of LINC01606 as a NC for promoter region and was also amplified by PCR using 5′‐biotin‐TEG‐labelled primer. The primer sequences were as follows: promoter region, forward primer: CGCTCTAGAGAGTCTGCTTTGAG; reverse primer, TATAGCGTTTCCCACCTGCC; NC region, forward primer: AGACTGACTTCCTCTTGAGGC; reverse primer, GCCCGCGCTCTTTAAAGATTATTA. SW480 cells were lysed, then nucleoprotein was extracted and quantified by standard procedures. The 40 μg of 5′‐biotin‐TEG‐labelled DNA probes were incubated for 30 min at room temperature in the presence of Dynabeads® C1 Streptavidin beads (Thermo Fisher) by the manufacturer's instructions to form DNA‐beads complexes. Then, the nucleoprotein was added to DNA–beads complexes and incubated overnight at 4°C to form DNA–protein–beads complexes. The complexes were decomposed in benzonase (Sigma–Aldrich) and trypsin (Promega, Madison, WI, USA), respectively. The eluted polypeptides were extracted and desalted by C18 columns (Sigma‐Aldrich), then polypeptides were identified using Q‐Exactive liquid chromatography tandem mass spectrometry (LC–MS/MS; Thermo Fisher). The MS data were analysed in MaxQuant software.

### 2.22 Bioinformatical analysis

The expression of lncRNA LINC01606, SCD1 in cancer tissues and normal tissues, TCGA dataset and correlation analysis were obtained from the DriverDBv3 database (websitehttp://driverdb.tms.cmu.edu.tw/),^32^ GEPIA website (http://gepia.cancer‐pku.cn/)^33^ and StarBase (http://starbase.sysu.edu.cn/index.php).^34^ The survival analysis was performed according to the DriverDBv3 database website^32^ and GEPIA website.^33^ The TFE3 Chip‐seq signal values on LINC01606 promoter region in A562 and HepG2 cells were predicted by ENCODE database website (https://www.encodeproject.org).^35^ The TFE3 immumohistochemical staining was obtained from The Human Protein Atlas website (https://www.proteinatlas.org/). JASPAR was used to predict sites of putative TFE3 binding motif in the LINC01606 promoter region (http://jaspar.genereg.net/).^36^

### 2.23 Statistical analyses

All statistical data analyses were performed with GraphPad Prism 9.0 (GraphPad Software, La Jolla, CA, USA) and the Statistical Program for Social Sciences 17.0 software (SPSS, Chicago, IL, USA). The values are presented as the mean ± standard deviation (SD) from at least three times of experiments. The differences between groups were assessed using Student's i‐test, one‐way analysis of variance (ANOVA), the Chi‐Square test or Fisher's exact test. All the tests were two‐tailed, and *p* < .05 was considered statistically significant (*p < .05, **p < .01, ***p < .001).

## 3 RESULTS

### 3.1 LINC01606 is frequently upregulated in colon cancer and correlates with poor outcomes in colon cancer patients

**FIGURE 1:**
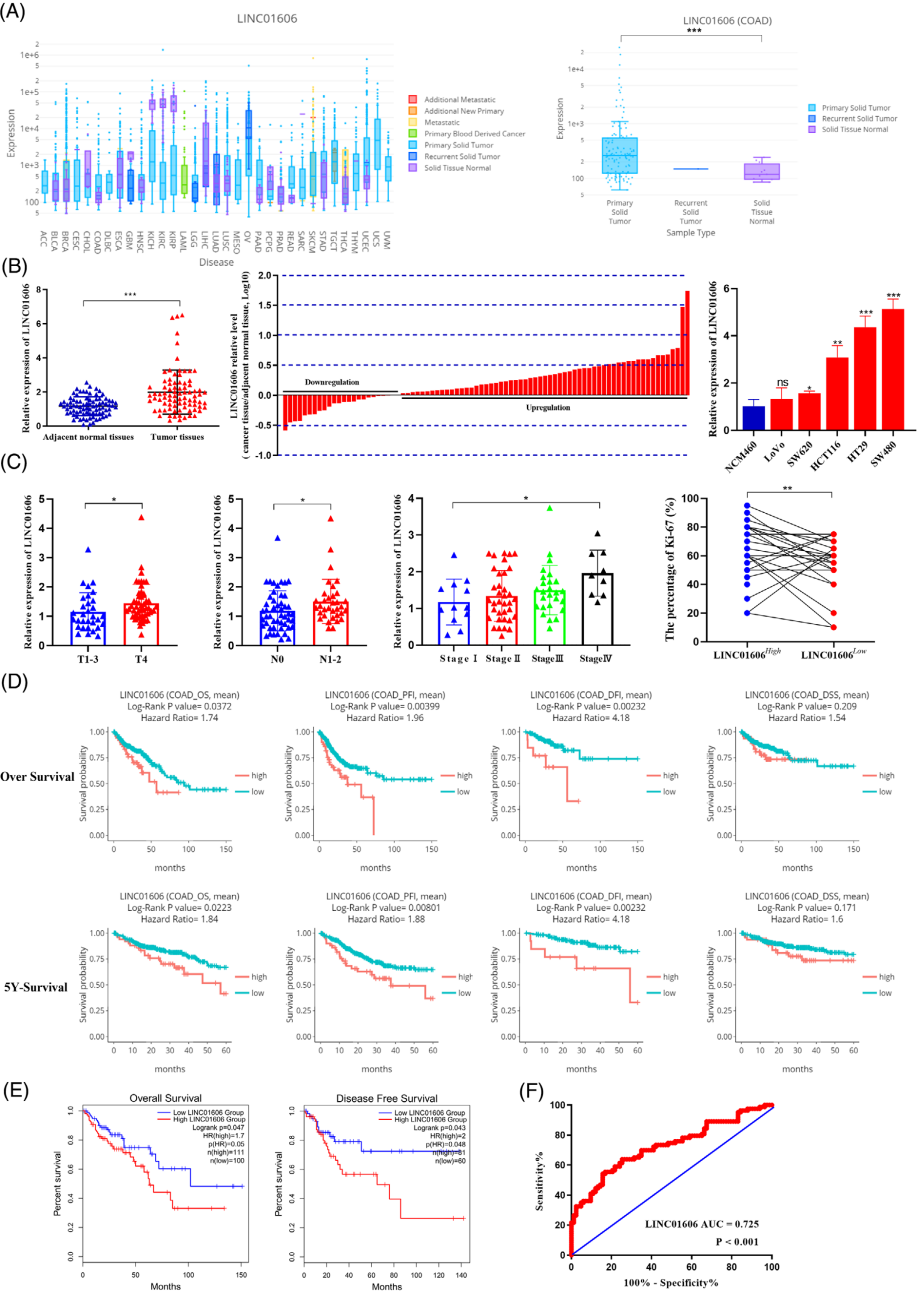
Expression level and prognostic value of LINC01606 in colon cancer. (A) LINC01606 expression level in pan‐cancer tissues and normal tissues. (B) LINC01606 expression levels were assessed in human colon cancer tissues compared with the paired adjacent normal tissues (*p* < .001, *n* = 83) and several human colon cancer cell lines compared with human normal colonic epithelial cell line. Colon cancer tissues express significantly higher levels of LINC01606 than paired adjacent normal tissues in the majority of patients (61 out of 83, 73.49%). Bars represent the ratio between expression in colon cancer tissue samples and adjacent normal tissue samples (C/N, log scale) from 83 patients was presented. Expression levels were normalised to GAPDH levels. (C) LINC01606 expression levels were assessed in human colon cancer tissues. The expression of LINC01606 were associated with depth of invasion (*n* = 83, *p* = .0474), lymph node metastasis (*n* = 83, *p* = .0477), stage (*n* = 83, *p* = .048) and Ki‐67 positive rate (*n* = 83, *p* = .004). Expression levels were normalised to GAPDH levels. (D) Kaplan–Meier estimates survival time of colon cancer patients in DriverDBv3 TCGA‐COAD cohort by different LINC01606 levels in tumour. High level of LINC01606 is associated with worse 5‐years and 12.5‐years overall survival (OS), progression‐free interval (PFI), disease‐free interval (DFI) and disease‐specific survival (DSS). (E) Kaplan–Meier estimates OS and disease free survival (DFS) of colon cancer patients in GEPIA. (F) Receiver operating characteristic (ROC) curve were performed to evaluate the diagnostic value of LINC01606 (*n* = 83, *p* = .048). (*p < .05, **p < .01 and ***p < .001). Abbreviations: ACC, adrenocortical carcinoma; BLCA, bladder urothelial carcinoma; BRCA, breast invasive carcinoma; CESC, cervical squamous cell carcinoma and endocervical adenocarcinoma; CHOL, cholangio carcinoma; COAD, colon adenocarcinoma; DLBC, lymphoid neoplasm diffuse large B‐cell lymphoma; ESCA, oesophageal carcinoma; GBM, glioblastoma multiforme; HNSC, head and neck squamous cell carcinoma; KICH, kidney chromophobe; KIRC, kidney renal clear cell carcinoma; KIRP, kidney renal papillary cell carcinoma; LAML, acute myeloid leukaemia; LGG, brain lower grade glioma; LIHC, liver hepatocellular carcinoma; LUAD, lung adenocarcinoma; LUSC, lung squamous cell carcinoma; MESO, mesothelioma; OV, ovarian serous cystadenocarcinoma; PAAD, pancreatic adenocarcinoma; PCPG, pheochromocytoma and paraganglioma; PRAD, prostate adenocarcinoma; READ, rectum adenocarcinoma; SARC, sarcoma; SKCM, skin cutaneous melanoma; STAD, stomach adenocarcinoma; TGCT, testicular germ cell tumours; THCA, thyroid carcinoma; THYM, thymoma; UCEC, uterine corpus endometrial carcinoma; UCS, uterine carcinosarcoma; UVM, uveal melanoma

The transcriptomic data from our previous study suggested that the lncRNA LINC01606 might be an oncology gene involved in the Wnt pathway and cancer progression. To test this possibility, we first evaluated LINC01606 expression in different cancer types, and the DriverDBv3 (http://driverdb.tms.cmu.edu.tw)‐TCGA database was utilised to identify mRNA expression levels. Analysis of RNA‐seq data from TCGA showed that LINC01606 was markedly upregulated in most cancer tissue types compared with normal tissues, such as BRCA, COAD, LUSC, READ and UCEC (*p *< .05; Figure 1(A)). In contrast, LINC01606 expression was suppressed in KICH, KIRC, KIRP and GBM (*p *< .05; Figure 1(A)). To further validate the expression of LINC01606 in colon cancer, 83 pairs of colon cancer samples and adjacent normal tissue samples, and several colon cancer cell lines and normal human colorectal epithelial cell line were validated by qRT‐PCR. Consistent with the TCGA data, LINC01606 was upregulated in colon cancer tissues compared with normal tissues and in colon cancer cell lines compared with normal colorectal epithelial cell line (Figure 1(B)). Furthermore, LINC01606 expression was higher in T4 cancer tissues than in T1 to T3 cancer tissues and in cancer tissues with the positive lymph node metastasis compared with negative lymph node metastasis (Figure 1(C)). Additionally, colon cancer patients in stage II, III and IV groups had relatively higher expression of LINC01606 than those in the stage I group (Figure 1(C)). Moreover, Ki‐67 expression was assessed based on LINC01606 expression. As expected, the positive percentage of Ki‐67 in cancer tissues with high LINC01606 expression was higher than that in cancer tissues with low LINC01606 expression (Figure 1(C)). Importantly, in DriverDBv3 database, the Kaplan–Meier survival analysis indicated that colon cancer patients with higher LINC01606 expression levels had a shorter 5‐year overall survival (OS) or total overall survival (5‐year‐OS: *p = *.0223, HR = 1.84; OS: *p *= .0372, HR = 1.74), progression‐free interval (5‐year‐PFI: *p* = .00801, HR = 1.88; PFI: *p *= .00399, HR = 1.96) and disease‐free interval (5‐year‐DFI: *p *= .00232, HR = 4.18; DFI: *p *= .00232, HR = 4.18) than those with lower LINC01606 expression levels. However, there were no significant differences between LINC01606 expression and disease‐specific survival (5‐year‐DSS: *p* = .171, HR = 1.60; DSS: *p *= .209, HR = 1.54) in colon cancer patients. Furthermore, colon cancer patients with higher LINC01606 expression levels also had a shorter OS (*p *= .047, HR = 1.70) and disease‐free survival (*p *= .043, HR = 2.0) in GEPIA (Figure 1(E)), further indicating that LINC01606 is most likely a biomarker for colon cancer.

To evaluate the clinical value of LINC01606 more comprehensively, receiver operating characteristic (ROC) curve analysis and relationships between LINC01606 expression and the clinicopathological characteristics were investigated. The area under the ROC curve (AUC) reached up to 0.725 (*p *< .001, 95% CI, 0.648 to 0.802), the sensitivity was 54.22% and the specificity was 84.34% with a Youden index of 0.386 in human colon cancer tissues (Figure 1(F)). Investigation of the correlation between LINC01606 expression and clinicopathological characteristics indicated that upregulation of LINC01606 was positively correlated with the positive rate of Ki‐67 (*p* = .005), depth of tumour invasion (*p* = .004), metastasis to lymph nodes (*p* = .028), advanced TNM stage (*p* = .038) and tumour venous/lymphatic/nervous invasion (*p* = .034) (Table 1). Thus, these analyses of the LINC01606 level and prognostic or diagnostic value in cancer specimens indicated that high expression of LINC01606 might play an oncogenic role and predict a poor prognosis in colon cancer.

**TABLE 1:** Association between the status of LINC010606 expression and clinicopathological characteristics of human colon cancer

		LINC01606 expression		
Characteristics	No. of cases	High	Low	*p* Value
All cases	83	61	22	
Age, (years, mean ± SD)	83	64.51 ± 12.56	64.45 ± 13.35	.987
BMI	83	22.79 ± 3.42	23.54 ± 2.94	.362
Tumour diameter (cm, mean ± SD)	83	5.24 ± 1.54	5.68 ± 2.19	.388
Ki‐67 (%, mean ± SD)	83	60.05% ± 18.58%	53.33% ± 19.64%	.005**
Gender				.568
Male	62	44	18	
Female	21	17	4	
Smoking				.291
Yes	21	14	7	
No	62	47	15	
Drinking alcohol				1.000
Yes	19	14	5	
No	64	47	17	
Depth of invasion				.004**
pT 1	2	1	1	
pT 2	10	5	5	
pT 3	20	11	9	
pT 4	51	44	7	
Lymph node metastasis				.028*
pN0	49	31	18	
pN1	23	21	2	
pN2	11	9	2	
Distant metastasis				.433
pM0	74	53	21	
pM1	9	8	1	
TNM stage	83			.038*
I	12	6	6	
II	36	24	12	
III	26	23	3	
IV	9	8	1	
Histology				.431
Poor differentiated	21	15	6	
Middle differentiated	56	40	16	
Well differentiated	6	6	0	
Venous/lymphatic/nervous invasion				.034*
Positive	27	24	3	
Negative	56	37	19	
Liver metastasis				1.000
Absent	78	57	21	
Present	5	4	1	
Fatty nodules				.094
Positive	22	13	9	
Negative	61	48	13	

BMI, body mass index.

^*^ *p *< .05.

^**^ *p* < .01.

### 3.2 LINC01606 promotes malignant phenotypes in colon cancer cells

**FIGURE 2:**
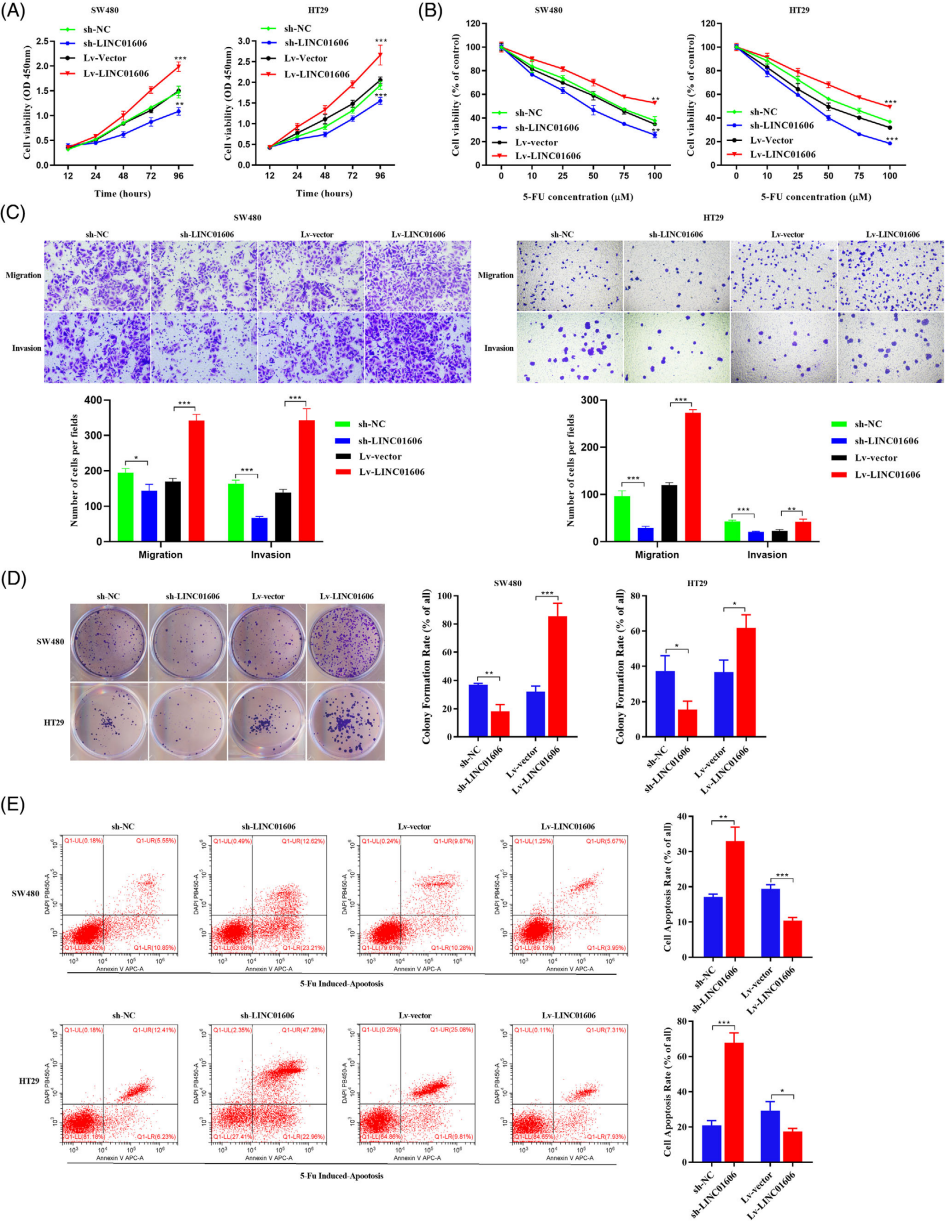
LINC01606 facilitates the progression of colon cancer cells. SW480 and HT29 cells are transfected with sh‐LINC01606‐expressing vector or LINC01606‐expressing vector (*n* = 3). (A) The cell proliferation assay was performed using the CCK‐8 assay (*n* = 3). (B) The cytotoxicity of 5‐Fu was performed using CCK‐ 8 assay (*n* = 3). (C) The migration and invasion of SW480 and HT29 cells were detected by Transwell assay (*n* = 3). Scale bars 10 μm. (D) The cell growth ability was assessed using colony formation. (E) The apoptosis of SW480 and HT29 cells detected by flow cytometry after treatment of 50 μM 5‐Fu (*n* = 3). Data are shown as the mean ± SD. *p < .05, **p < .01 and ***p < .001 compared with control

To investigate the biological function of LINC01606 in colon cancer cells, we generated SW480 and HT29 clones that had either stable knockdown of LINC01606 by shRNALINC01606 or stable overexpression of LINC01606 transcript by lentivirus. The efficiency of stable knockdown and overexpression of LINC016016 was detected by qRT–PCR (Figure S1). We performed cell proliferation, cytotoxicity, colony formation, transwell invasion or migration and apoptosis assays to confirm the malignant phenotypes we observed after stable knockdown or overexpression of LINC016016. Silencing of LINC01606 resulted in a strong antiproliferative effect in SW480 and HT29 cells (Figure 2(A)). In contrast, the proliferative ability of colon cancer cells was increased by overexpression of LINC016016 (Figure 2(A)). Moreover, the cell proliferation ability was significantly inhibited after 5‐Fu treatment, with more dramatic inhibition observed in sh‐LINC01606 cells compared with sh‐NC cells and less remarkable inhibition observed in Lv‐LINC01606 cells compared with empty vector (Lv‐Vector) cells or sh‐LINC01606 cells (Figure 2(B)). Similarly, transwell invasion and migration and colony formation assays showed that LINC01606 inhibition significantly inhibited cell migration and invasion compared with control cells. Conversely, overexpression of LINC016016 significantly promoted cell migration and invasion (Figures 2(C) and 2(D)). Moreover, flow cytometric analysis was performed to further address whether LINC01606 affected colon cancer cell apoptosis after 5‐Fu treatment. The results revealed that LINC01606 knockdown markedly increased the proportion of apoptotic cells compared with sh‐NC cells, and LINC01606 overexpression significantly decreased the proportion of apoptotic cells compared with Lv‐Vector cells (Figure 2(E)). Together, our functional assays demonstrate that LINC01616 promotes malignant phenotypes by enhancing cell growth, invasion and anti‐apoptosis in vitro.

### 3.3 LINC01606 enhances CSC properties in colon cancer cells

**FIGURE 3:**
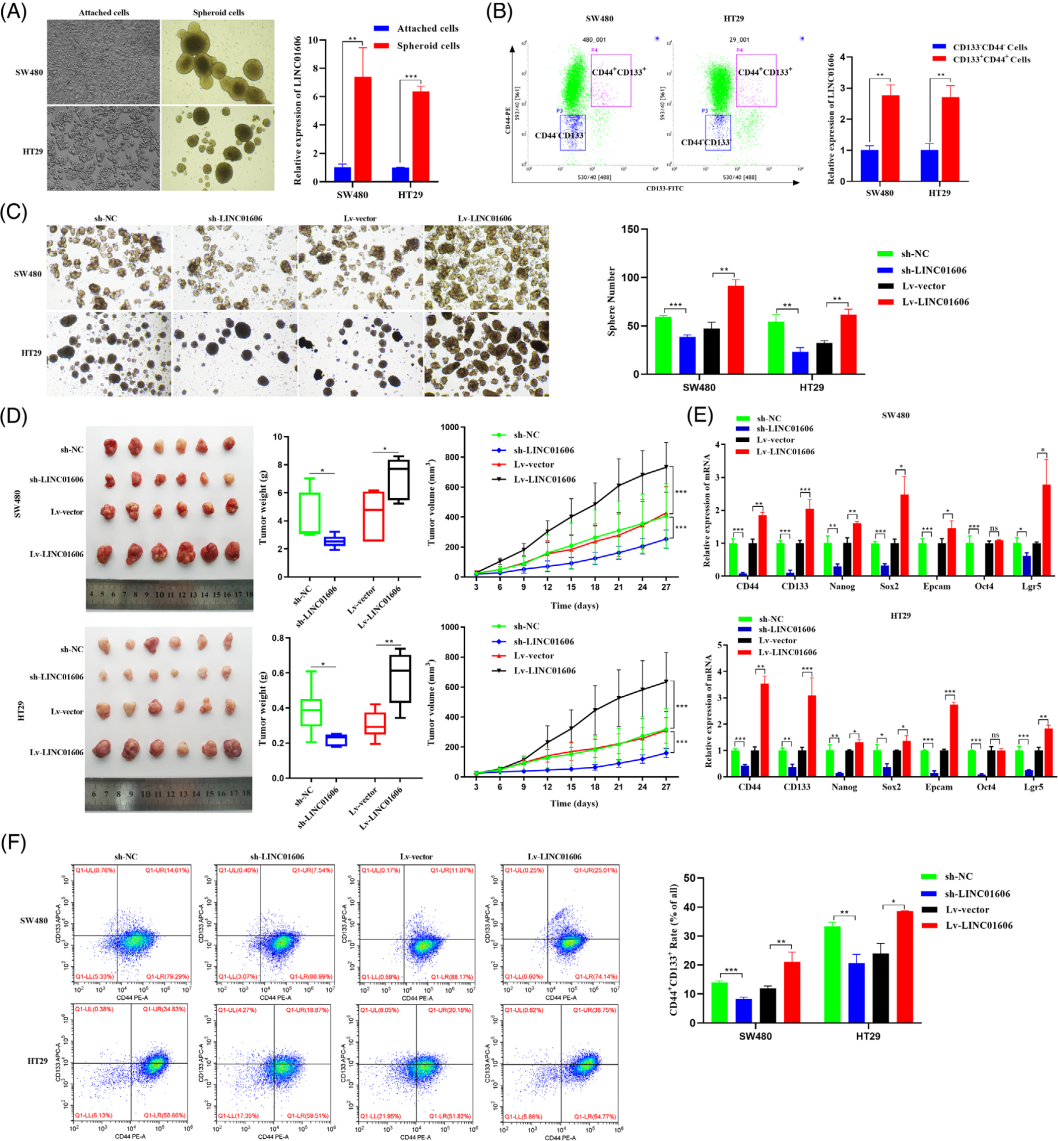
LINC01606 enhances the stemness of colon cancer cells. (A) Representative images of adherent cells and the tumour spheres. The expression level of LINC01606 in adherent cells and tumour spheres was detected by qRT‐PCR (*n* = 3). (B) Cells were double stained with anti‐CD44‐PE and anti‐CD133‐FITC. Flow cytometry fluorescent cell sorting was used to attain CD44+CD133+ and CD44−CD133− colon cancer cells. The expression level of LINC01606 in CD44+CD133+ and CD44−CD133− colon cancer cells was detected by qRT‐PCR (*n* = 3). (C) Tumour sphere formation of SW480 and HT29 cells after transfected with sh‐ LINC01606‐expressing vector or LINC01606‐expressing vector. (D) Representative images of tumours in xenograft mouse models bearing SW480 and HT29 cells transfected with sh‐LINC01606‐expressing vector or LINC01606‐expressing vector. The tumour volumes were measured every 3 days and the tumour weights were measured after 4 weeks when mice were sacrificed (*n* = 6). (E) The effect of LINC01606 on colon cancer cell stem‐like markers. CD44, CD133, Nanog, Sox2, Epcam, Oct4, and Lgr5 levels were analysed by qRT‐PCR after transfected with LINC01606 shRNA‐expressing vector or LINC01606‐expressing vector (*n* = 3). (F) Flow cytometry analysis of the quantity of CD44+CD133+ cells in SW480 and HT29 after transfected with LINC01606 shRNA‐expressing vector or LINC01606‐expressing vector (*n* = 3). Data are shown as the mean ± SD. *p < .05, **p < .01 and ***p < .001 compared with control

The formation of tumour spheres is a major characteristic of both normal stem cells and CSCs. To validate the potential role of LINC01606 in human colon cCSCs, we detected LINC01606 expression in SW480 and HT29 adherent cells and tumour spheres by qRT‐PCR. LINC01606 expression was substantially upregulated in both tumour spheres compared with adherent cells (Figure 3(A)). CD133^+^CD44^+^ cells have CSC characteristics and CD133^−^CD44^−^ cells have regular cancer cell characteristics. Therefore, we sorted CD133^−^CD44^−^ cells and CD133^+^CD44^+^ cells from SW480 and HT29 cells and then detected the expression level of LINC01606. Compared with CD133^−^CD44^−^ cells, LINC01606 expression was substantially higher than that in CD133^+^CD44^+^ cells (Figure 3(B)). To further confirm that LINC01606 expression affected CSC‐related properties, we generated LINC01606‐silenced and LINC01606‐overexpressing SW480 and HT29 cells for sphere formation in vitro and a xenograft model in vivo. As expected, the number of spheres formed was significantly inhibited in response to LINC01606 depletion, and conversely, LINC01606 overexpression significantly increased the tumour sphere number (Figure 3(C)). Assays in a mouse xenograft model showed that subcutaneous xenografts derived from SW480 and HT29 cells overexpressing LINC01606 had significantly increased tumour weights, volumes and sizes after 1 month of growth, whereas xenografts derived from SW480 and HT29 cells with LINC01606 silencing had significantly reduced tumour weights, volumes and sizes after 1 month of growth compared with each control (Figure 3(D)); the whole‐body weight remained unchanged (Figure S2).

To determine whether LINC01606 knockdown affected stemness‐related markers in colon cancer cells, we performed shRNA knockdown of LINC01606 and lentivirus overexpression of LINC01606 in SW480 and HT29 cells followed by analysis of established CSC markers. We found that LINC016606 depletion was associated with significantly decreased mRNA expression of colon carcinogenesis and CSC‐related markers, including CD133, CD44, Nanog, Epcam, Sox2, Oct4 and Lgr5; and LINC016606 overexpression was associated with significantly increased mRNA expression of CD133, CD44, Nanog, Epcam, Sox2, Oct4 and Lgr5, but the expression of Oct4 was no significant difference (Figure 3(E)). Furthermore, flow cytometry analysis of the percentage of CD133^+^CD44^+^ cells in SW480 and HT29 cells was performed. LINC01606 knockdown significantly reduced the percentage of CD133^+^CD44^+^ cells; in contrast, LINC01606 overexpression significantly increased the percentage of CD133^+^CD44^+^ cells in these two transfected cell lines (Figure 3(F)). These results clearly demonstrated that LINC01606 performed a critical oncogenic function that promoted the stemness of colon cancer cells.

### 3.4 LINC01606 regulates SCD1 expression by competing for miR‐423‐5p at the transcription level

**FIGURE 4:**
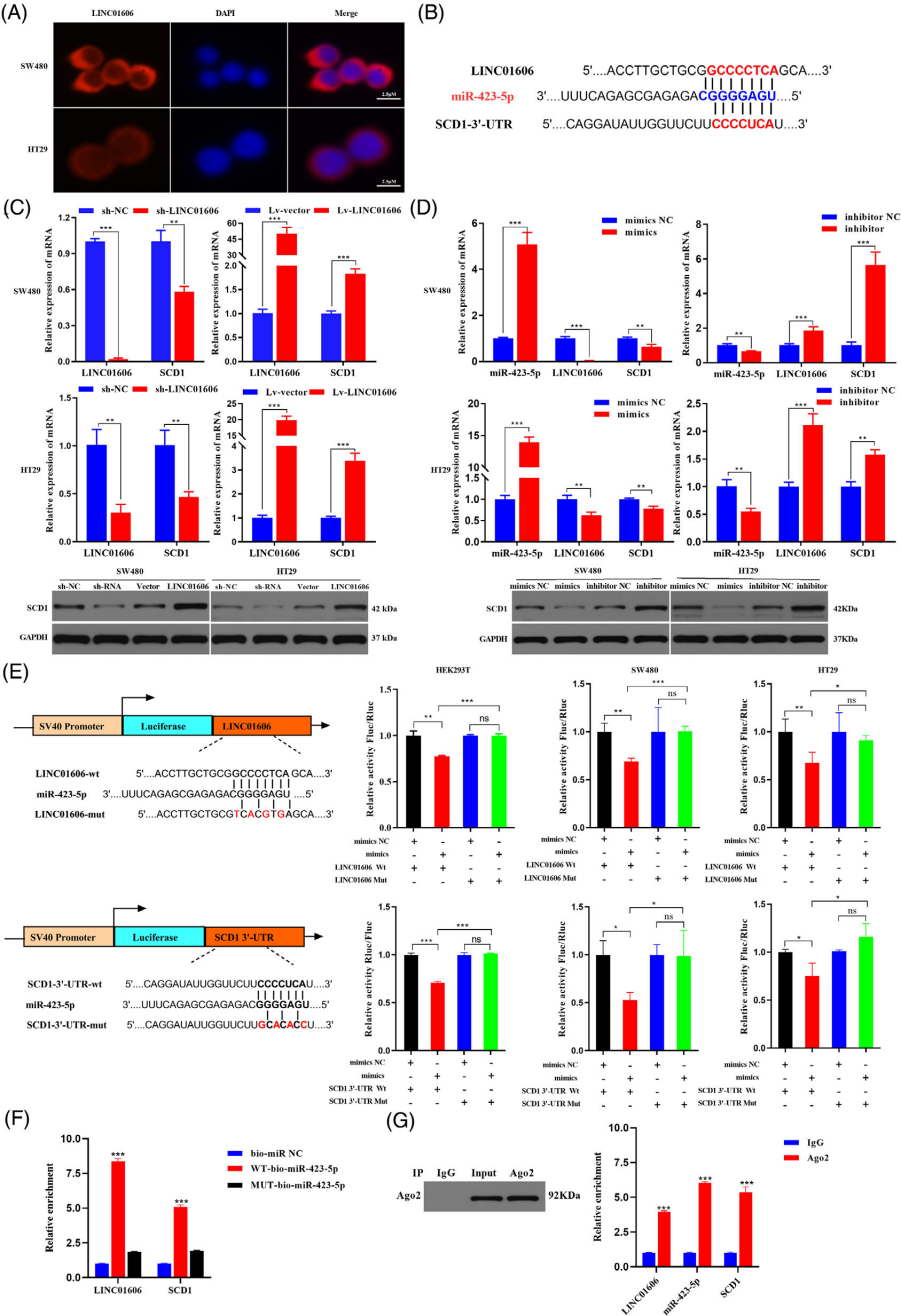
LINC01606 affected the SCD1 level by interacting with miR‐423‐5p. (A) The location of LINC01606 in colon cancer cells by FISH assays. Scale bars 2.5 μm. (B) The binding sites of miR‐423‐5p on LINC01606 and SCD1 3′UTR. (C) Protein and mRNA expression level of SCD1 in SW480 and HT29 cells after transfection with sh‐LINC01606‐expressing vector or LINC01606‐expressing vector (*n* = 3). (D) mRNA expression level of LINC01606 and protein and mRNA expression level of SCD1 in SW480 and HT29 cells after transfection with miR‐423‐5p mimics or inhibitors (*n* = 3). (E) Luciferase reporting gene assay was performed to analyse whether miR‐423‐5p could bind to LINC01606 and SCD1 3′UTR (*n* = 3). (F and G) Interaction of miR‐423‐5p with lncRNA LINC01606 and SCD1 was investigated through the RIP and pull‐down assays in SW480 (*n* = 3). Data are shown as the mean ± SD. ns, no significant (*p* > .05), *p < .05, **p < .01 and ***p < .001 compared with control

Our previous studies indicated that LINC01606 promoted Wnt3a expression by competing for miR‐423‐5p.^31^ To validate the miR‐423‐5p physical interaction between LINC01606 and the Wnt3a 3′UTR in colon cancer, we primarily performed FISH to confirm the subcellular location of LINC01606 in SW480 and HT29 cells. Subcellular localisation assays revealed that LINC01606 was mainly located in the cytoplasm, while a less positive signal was also observed in the nucleus (Figure 4(A)). Subsequently, overexpression of LINC01606 significantly enhanced Wnt3a expression in SW480 and HT29 cells, whereas the knockdown of LINC01606 significantly inhibited Wnt3a expression in these two cell lines (Figure S3(A)). Next, we examined whether miR‐423‐5p inhibited LINC01606 and Wnt3a expression. We found that overexpression of miR‐423‐5p significantly inhibited LINC01606 expression by transfection of miR‐423‐5p mimics, whereas a sponge of miR‐423‐5p increased LINC01606 expression by transfection of miR‐423‐5p inhibitors (Figure 4(D)). Consistently, overexpression or depletion of miR‐423‐5p could significantly inhibited or increased Wnt3a, respectively (Figure S3(A)). To test the functional results of miR‐423‐5p binding to LINC01606 and the Wnt3a 3′UTR, the predicted miR‐423‐5p binding site of LINC01606‐WT or Wnt3a 3′UTR‐WT and a mutated miR‐423‐5p binding site of LINC01606 mutant type (LINC01606‐MUT) or Wnt3a 3′UTR mutant type (Wnt3a 3′UTR‐MUT) were cloned into luciferase reporter plasmids. It was observed that miR‐423‐5p significantly decreased luciferase activity in the LINC01606 WT vector but not in the LINC01606 mutant vector following transfection of miR‐423‐5p, LINC01606‐WT or LINC01606‐MUT vector (Figure 4(E)). Interestingly, we found that miR‐423‐5p could not decrease luciferase activity in either the Wnt3a 3′UTR WT vector or the Wnt3a 3′UTR mutant vector (Figure S3(B)). These findings indicated that miR‐423‐5p could significantly suppress Wnt3a expression indirectly instead of by direct physical binding to Wnt3a 3′UTR sites.

Based on the above findings, we hypothesised that LINC01606 might regulate the region upstream of Wnt3a to cause changes in its expression. SCD1 is an enzyme that is required to produce active, lipid‐modified WNT proteins.^37^ According to TargetScan tool analysis, we found that the SCD1 3′UTR sequences also contained a miR‐423‐5p binding site (Figure 4(B)). To better understand the relationship between LINC01606 and SCD1 in colon cancer, we first addressed SCD1 expression in 83 colon cancer patients. The results revealed that SCD1 expression was significantly increased in colon cancer tissues when compared with paired adjacent normal tissues (Figure S4(A)). Similarly, SCD1 expression was also significantly increased in colon cancer tissues compared with normal tissues from the TCGA database (Figure S4(B)). Moreover, significantly higher levels of SCD1 were observed in cancerous tissues with high LINC01606 expression than in cancerous tissues with low LINC01606 expression (Figure S4(C)). Subsequently, we found a strong correlation between LINC01606 and SCD1 expression in 83 colon cancer patients (Figure S4(D)), suggesting that LINC01606 might promote SCD1 expression.

To validate whether miR‐423‐5p had a physical interaction with the SCD1 3′UTR in colon cancer, we first detected SCD1 expression following interference or overexpression of LINC01606 and miR‐423‐5p in SW480 and HT29 cells. We found that depletion of LINC01606 or overexpression of miR‐423‐5p significantly inhibited SCD1 expression, whereas overexpression of LINC01606 or depletion of miR‐423‐5p increased SCD1 expression (Figures 4(C) and 4(D)). Then, we performed a Dual‐Luciferase reporter assay, and the results indicated lower luciferase activity in the SCD1 3′UTR‐WT + miR‐423‐5p mimics group than in the SCD1 3′UTR‐WT + miR‐423‐5p mimics NC group and the SCD1 3′UTR‐MUT + miR‐423‐5p mimics group (Figure 4(E)). qRT‐PCR results confirmed the enrichment of LINC01606 and SCD1 in the compounds pulled down by the biotin‐labelled WT‐bio‐miR‐423‐5p sequences rather than by the biotin‐labelled MUT‐bio‐miR‐423‐5p probe sequences (Figure 4(F)). The RIP of LINC01606, miR‐423‐5p and SCD1 by Ago2 indicated that miR‐423‐5p potentially interacted with LINC01606 or SCD1 in RNA‐induced silencing complexes (Figure 4(G)). Taken together, these findings indicated that LINC01606 enhances SCD1 expression in colon cancer by competing for miR‐423‐5p.

### 3.5 LINC01606 protects colon cancer cells from ferroptotic cell death

**FIGURE 5:**
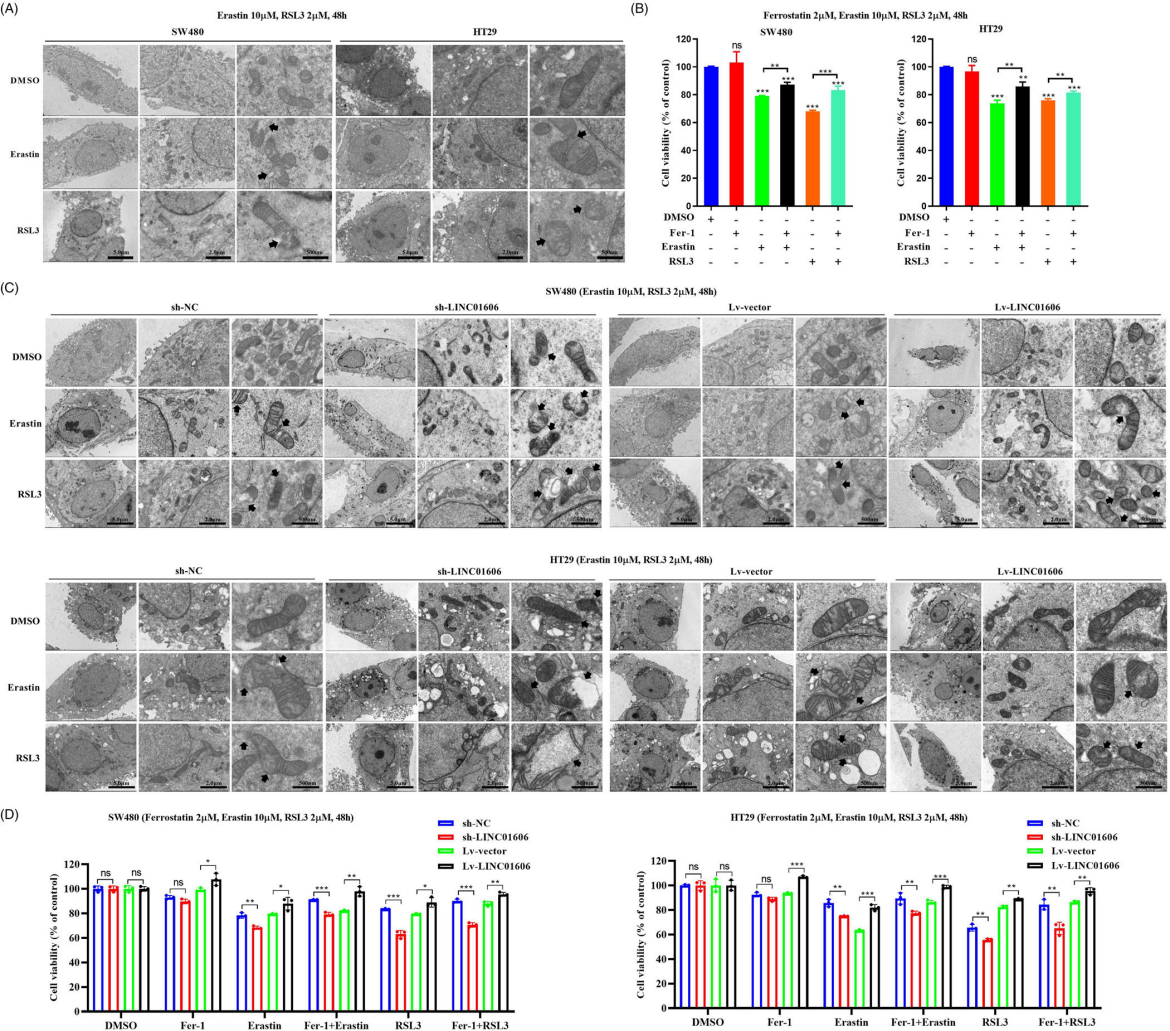
Morphological changes of ferroptosis in colon cancer cells. (A) Transmission electron microscope images of mitochondria in SW480 and HT29 cells after treatment of DMSO, Erastin (10 μM) and RSL3 (2 μM) for 48 h. The black arrows indicate the outer mitochondrial membrane was ruptured and the mitochondrial cristae decreased or disappeared. Scale bars 5 μm, 2 μm and 100 nm. (B) A CCK‐8 assay was used to assess cell viability in SW480 and HT29 cells after treatment of DMSO, Ferrostatin‐1 (Fer‐1, 10 μM), Erastin (10 μM) and RSL3 (2 μM) for 48 h (*n* = 3). (C) Representative transmission electron microscopy images of mitochondria in LINC01606 stable knockdown or LINC01606 stable overexpression SW480 and HT29 cells after treatment of DMSO, Erastin (10 μM) and RSL3 (2 μM) for 48 h. Scale bars 5 μm, 2 μm and 100 nm. The black arrows indicate the outer mitochondrial membrane of neurons was ruptured and the mitochondrial cristae decreased or disappeared. Scale bars 5 μm, 2 μm and 100 nm. (D) A CCK‐8 assay was used to assess cell viability in LINC01606‐knowdown or LINC01606‐overexpressed SW480 and HT29 cells after treatment of DMSO, Ferrostatin‐1 (Fer‐1, 10 μM), Erastin (10 μM) and RSL3 (2 μM) for 48 h (*n* = 3). Data are shown as the mean ± SD. ns, no significant (*p* > .05), *p < .05, **p < .01 and ***p < .001 compared with control

Recent advances have indicated that SCD1 inhibits ferroptotic cell death through decreasing intracellular lipid ROS.^38,39^ It is worth noting that SCD1 is a target gene of LINC01606, and we wondered whether LINC01606 also had a suppressive role in ferroptosis in colon cancer cells. Therefore, we first performed transmission electron microscopy and cell viability after treatment of colon cancer cells using Erastin or RSL3, inducers of ferroptosis, which preliminarily confirmed that ferroptosis could occur in SW480 and HT29 colon cancer cells. Preliminary evidence showed that the Erastin‐induced or RSL3‐induced morphological features of mitochondria involved membrane rupture, a smaller size, a decrease in or disappearance of cristae and an increase in membrane density (Figure 5(A)), and Erastin‐induced or RSL3‐induced cell growth inhibition. Fer‐1, an inhibitor of ferroptosis, reversed those induced cell growth inhibition in SW480 and HT29 cells (Figure 5(B)). Subsequently, we explored LINC01606 expression in Erastin‐induced or RSL3‐induced ferroptotic morphological changes and cell growth inhibition. Knockdown of LINC01606 in SW480 and HT29 cells significantly increased Erastin‐induced or RSL3‐induced rupture of the mitochondrial membrane and the disappearance of mitochondrial cristae and significantly enhanced Erastin‐induced ferroptotic cell growth inhibition, whereas overexpression of LINC01606, likely Fer‐1, had a slight impact on Erastin‐induced or RSL3‐induced mitochondrial morphology and cell growth inhibition (Figures 5(C) and 5(D)). Consequently, LINC01606 might suppress the occurrence of ferroptosis in colon cancer cells.

**FIGURE 6:**
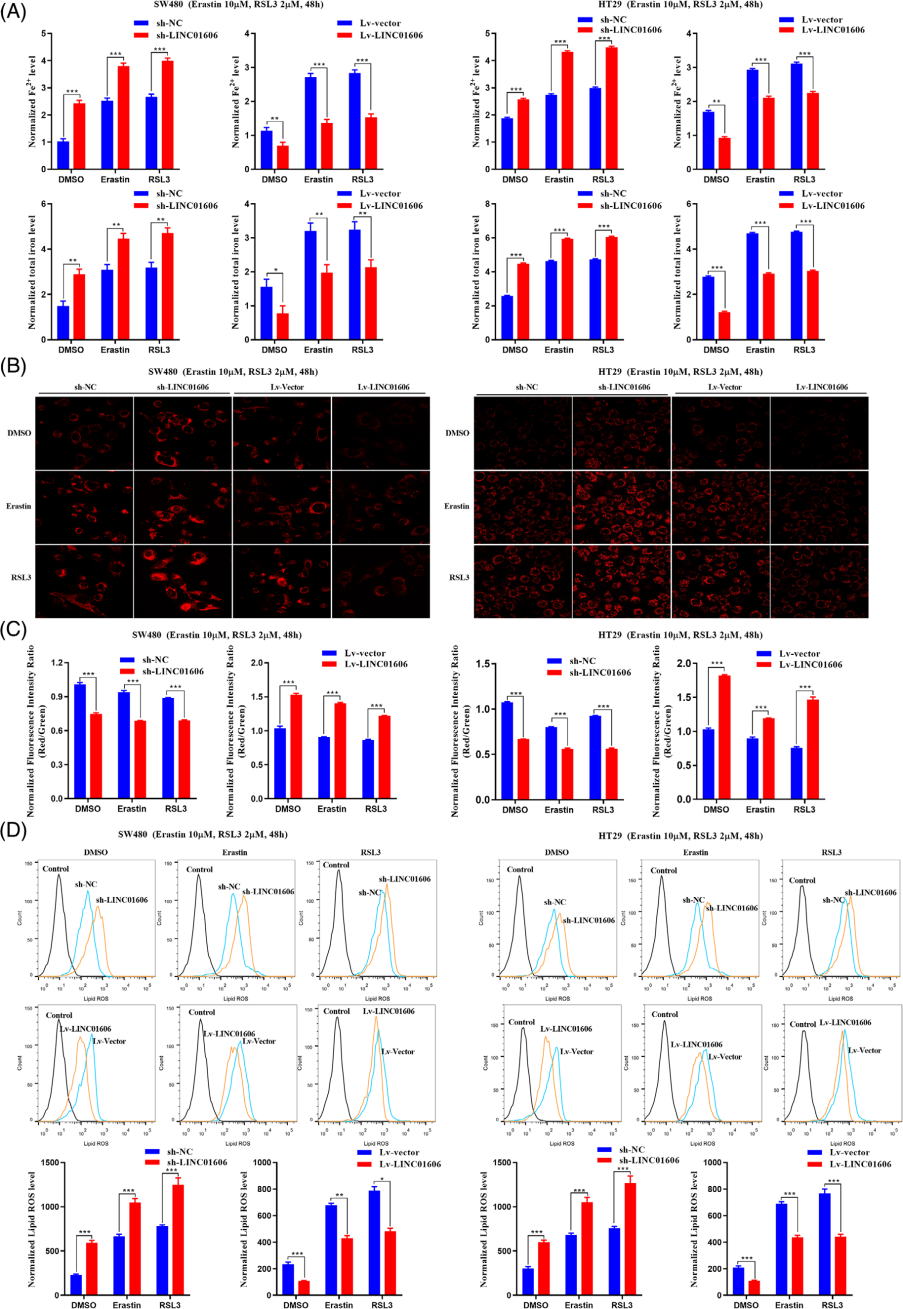
LINC01606 suppresses colon cancer cell ferroptosis. (A) Levels of intracellular total iron and ferrous iron (Fe^2+^) were analysed in SW480 and HT29 cells subjected to the stable knockdown of LINC001606 or stably overexpressing LINC001606 after treatment of DMSO, Erastin (10 μM) and RSL3 (2 μM) for 48 h (*n* = 3). (B) Representative images of intracellular mitochondrial superoxide level in SW480 and HT29 cells subjected to the stable knockdown of LINC001606 or stably overexpressing LINC001606 after treatment of DMSO, Erastin (10 μM) and RSL3 (2 μM) for 48 h. Scale bars 2.5 μm (*n* = 3). (C) Levels of mitochondrial membrane potential were analysed in SW480 and HT29 cells subjected to the stable knockdown of LINC001606 or stably overexpressing LINC001606 after treatment of DMSO, Erastin (10 μM) and RSL3 (2 μM) for 48 h (*n* = 3). (D) Flow cytometry analysis the relative levels of intracellular lipid ROS in SW480 and HT29 cells subjected to the stable knockdown of LINC001606 or stably overexpressing LINC001606 after treatment of DMSO, Erastin (10 μM) and RSL3 (2 μM) for 48 h (*n* = 3). Data are shown as the mean ± SD. *p < .05, **p < .01 and ***p < .001 compared with control

To further explore the role of LINC01606 in Erastin‐ or RSL3‐involved colon cancer cell ferroptosis, we measured intracellular levels of total iron, Fe^2+^, mitochondrial superoxide, mitochondrial membrane potential and lipid ROS following treatment with Erastin and RSL3 in LINC01606 knockdown or overexpression SW480 and HT29 cells. As a result, we found that knockdown of LINC01606 expression levels significantly enhanced intracellular concentrations of total iron and Fe^2+^ (Figure 6(A)), mitochondrial superoxide (Figure 6(B)) and lipid ROS (Figure 6(D)) and decreased the mitochondrial membrane potential (Figure 6(C)) in SW480 and HT29 cells, indicating that the depletion of LINC01606 induced sensitivity to ferroptosis. Furthermore, overexpression of LINC01606 significantly decreased intracellular concentrations of total iron and Fe^2+^, mitochondrial superoxide and lipid ROS and increased mitochondrial membrane potential in SW480 and HT29 cells (Figures 6(A)–6(D)), indicating that the overexpression of LINC01606 induced resistance to ferroptosis. Together, these findings further demonstrated that LINC01606 not only inhibited cell apoptosis but also suppressed ferroptosis.

### 3.6 LINC01606 and Wnt/β‐catenin signalling forms a positive feedback regulatory loop

**FIGURE 7:**
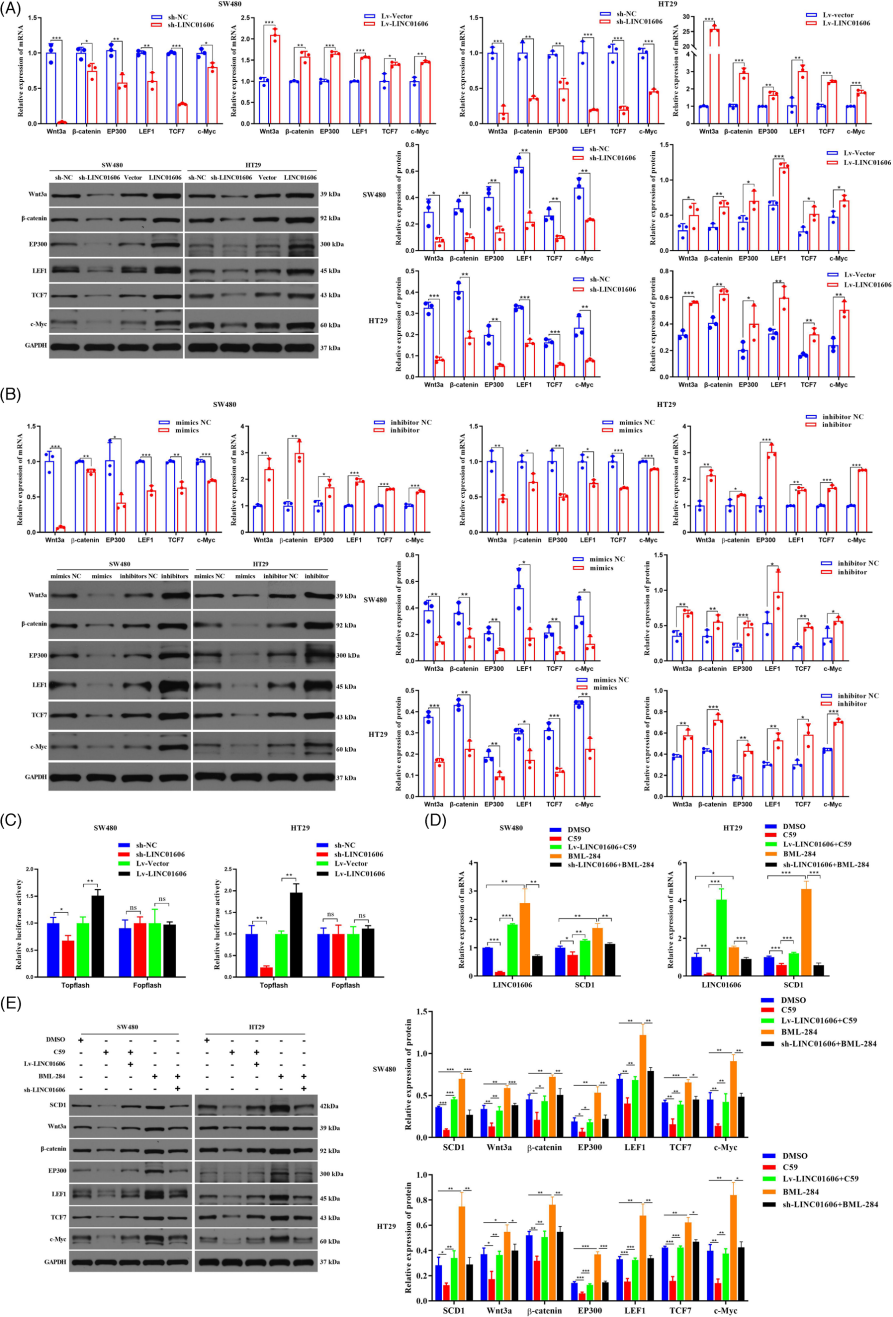
LINC001606 promotes Wnt signalling activity. (A) The mRNA and protein level of Wnt/β‐catenin signalling moleculars including: Wnt3a, β‐catenin, Ep300, LEF1, TCF7 and c‐Myc in SW480 and HT29 cells subjected to the stable knockdown of LINC001606 or stably overexpressing LINC001606 (*n* = 3). (B) The mRNA and protein level of Wnt/β‐catenin signalling moleculars including: Wnt3a, β‐catenin, Ep300, LEF1, TCF7 and c‐Myc in SW480 and HT29 cells subjected to depletion of miR‐423‐5p or overexpressing miR‐423‐ 5p (*n* = 3). (C) The luciferases activity was designed to detect β‐catenin‐mediated TCF/LEF transcription activity by TOPFlash/FOPFlash system assay in SW480 and HT29 cells after transfection with sh‐LINC01606‐ expressing vector or LINC01606‐expressing vector. The luciferase activity with sh‐LINC01606 was divided by that with control sh‐NC or with Lv‐LINC01606 was divided by that with control vector (*n* = 3). (D) The mRNA level of LINC01606 and SCD1 in SW480 and HT29 cells transfected with sh‐LINC01606‐expressing vector or LINC01606‐expressing vector after treatment of 10 μM Wnt signalling inhibitor C59 and 0.5 μM Wnt signalling activator BML‐284 for 48 h (*n* = 3). (E) The protein level of SCD1 and Wnt/β‐catenin signalling moleculars in SW480 and HT29 cells transfected with sh‐LINC01606‐expressing vector or LINC01606‐ expressing vector after treatment of 10 μM Wnt signalling inhibitor C59 and 0.5 μM Wnt signalling activator BML‐284 for 48 h (*n* = 3). Data are shown as the mean ± SD. *p < .05, **p < .01 and ***p < .001 compared with control

The production of a lipid‐modified WNT protein by SCD1, a target gene of LINC01606, is a vital step in the activation of the Wnt/β‐catenin signalling. To investigate whether LINC001606 could regulate the Wnt/β‐catenin signalling, we first examined the effect of LINC001606 on downstream molecules of the Wnt/β‐catenin signalling in colon cancer cells. The results revealed that molecules involved in Wnt/β‐catenin signalling, including Wnt3a, β‐catenin, Ep300, LEF1, TCF7 and c‐Myc, were markedly decreased after knockdown of LINC01606, whereas they were markedly increased after overexpression of LINC01606 in SW480 and HT29 cells (Figure 7(A)). Subsequently, we assessed the impact of miR‐423‐5p on Wnt/β‐catenin signalling in colon cancer cells. When miR‐423‐5p was depleted, the Wnt/β‐catenin signalling components were markedly increased. Correspondingly, overexpression of miR‐423‐5p could markedly decreased Wnt/β‐catenin signalling components in SW480 and HT29 cells (Figure 7(B)).

Next, to further investigate the impact of LINC01606 on the Wnt signalling pathway, we performed a TOPFlash/FOPFlash system assay. TOPFlash is an improved LEF/TCF reporter plasmid that was developed with repeated LEF/TCF‐binding motifs and a versatile luciferase gene, whereas FOPFlash is a control plasmid containing mutated TCF/LEF‐binding motifs that is widely used in detecting β‐catenin‐mediated LEF/TCF transcription activity in the Wnt signalling pathway. The results demonstrated that β‐catenin‐mediated LEF/TCF transcription activity was significantly increased in LINC001606‐overexpressing SW480 and HT29 cells, whereas β‐catenin‐mediated LEF/TCF transcription activity was significantly decreased in LINC001606‐knockdown SW480 and HT29 cells (Figure 7(C)), indicating that LINC001606 activated Wnt signalling through β‐catenin‐mediated LEF/TCF transcription activity.

Furthermore, we investigated the relationship between LINC001606 expression and Wnt signalling activity by treating colon cancer cells with the Wnt signalling inhibitor C59 and the Wnt signalling activator BML‐284. Interestingly, we found that C59 not only significantly inhibited Wnt signalling but also significantly decreased LINC001606 and SCD1 expression; BML‐284 not only significantly activated Wnt signalling but also significantly enhanced LINC001606 and SCD1 expression in SW480 and HT29 cells (Figures 7(D) and 7(E)). Furthermore, the sh‐LINC01606 downregulation effect was partly abolished by treatment with BML‐284, and the Lv‐LINC01606 upregulation effect was also partly abolished by treatment with C59 in SW480 and HT29 cells (Figures 7(D) and 7(E)). Based on the above results, we inferred that LINC01606 could activate the activity of the Wnt/β‐catenin signalling, whereas activated Wnt/β‐catenin signalling could promote LINC01606 expression, which formed a positive feedback regulatory loop in colon cancer cells.

### 3.7 LINC01606‐Wnt/β‐catenin positive regulatory signalling and colon CSCs block ferroptosis

**FIGURE 8:**
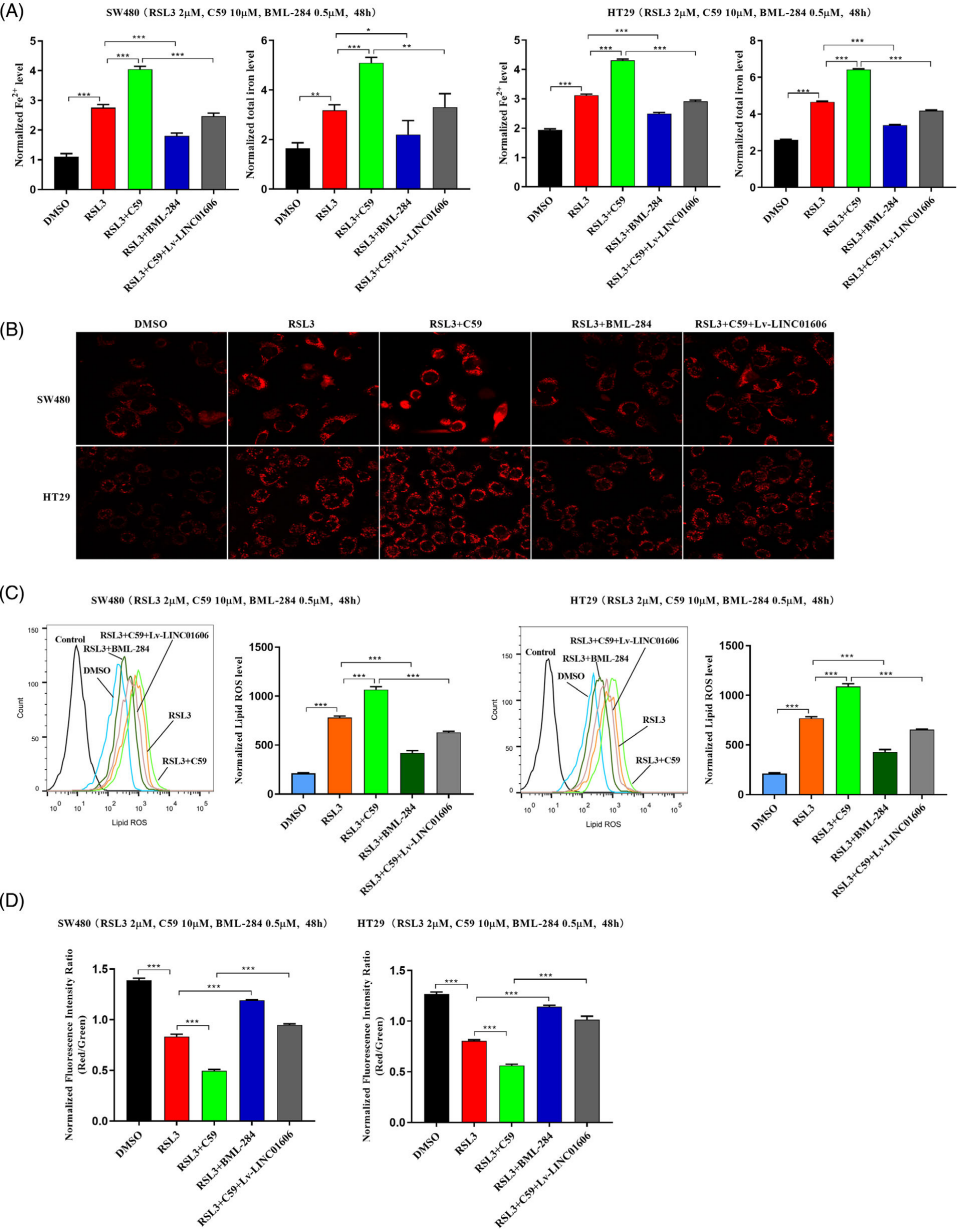
LINC01606–Wnt/β‐catenin axis suppresses ferroptosis in colon cancer cells. (A) Levels of intracellular total iron and Fe^2+^ were analysed in SW480 and HT29 cells subjected to overexpressing LINC001606 or treatment of DMSO, C59 (10 μM), BML‐284 (0.5 μM) and RSL3 (2 μM) for 48 h (*n* = 3). (B) Representative images of intracellular mitochondrial superoxide level in SW480 and HT29 cells subjected to overexpressing LINC001606 or treatment of DMSO, C59 (10 μM), BML‐284 (0.5 μM) and RSL3 (2 μM) for 48 h (*n* = 3). Scale bars 2.5 μm. (C) Flow cytometry analysis the relative levels of intracellular lipid ROS in SW480 and HT29 cells subjected to overexpressing LINC001606 or treatment of DMSO, C59 (10 μM), BML‐ 284 (0.5 μM) and RSL3 (2 μM) for 48 h. (D) Levels of mitochondrial membrane potential were analysed in SW480 and HT29 cells subjected to overexpressing LINC001606 or treatment of DMSO, C59 (10 μM), BML‐ 284 (0.5 μM) and RSL3 (2 μM) for 48 h (*n* = 3). Data are shown as the mean ± SD. *p < .05, **p < .01 and ***p < .001 compared with control

Given that LINC01606 and SCD1 are essential in the maintenance of colon CSCs and that both participate in the activation of the Wnt/β‐catenin signalling, we sought to determine whether the LINC01606‐Wnt/β‐catenin signalling and colon CSCs were involved in Erastin‐ or RSL3‐induced ferroptosis in colon cancer cells. We first explored the role of the LINC01606–Wnt/β‐catenin axis in the process of RSL3‐induced ferroptosis by treatment with the Wnt signalling inhibitor C59 or Wnt signalling activator BML‐284 combined with LINC01606 overexpression. The results showed that blocking LINC01606–Wnt/β‐catenin axis signalling sensitised colon cancer cells to the ferroptosis inducer RSL3 by C59 treatment of SW480 and HT29 cells. The intracellular concentrations of total iron and Fe^2+^ (Figure 8(A)), mitochondrial superoxide (Figure 8(B)) and lipid ROS (Figure 8(C)) were significantly increased in the C59 and RSL3 treatment group compared with the RSL3 group, whereas the mitochondrial membrane potential was significantly decreased (Figure 8(D)). In contrast, activation of LINC01606–Wnt/β‐catenin axis signalling led to resistance of colon cancer cells to the ferroptosis inducer RSL3 by BML‐284 treatment of SW480 and HT29 cells. The intracellular concentrations of total iron and Fe^2+^ (Figure 8(A)), mitochondrial superoxide (Figure 8(B)) and lipid ROS (Figure 8(C)) were significantly decreased in the BML‐284 and RSL3 treatment group compared with the RSL3 group, whereas the mitochondrial membrane potential was significantly increased (Figure 8(D)). Furthermore, the above C59 blocking effects were alleviated in the rescue group by overexpression of LINC01606 (Figures 8(A)–8(D)). Taken together, these findings confirmed that activation of LINC01606–Wnt/β‐catenin axis signalling significantly attenuated RSL3‐induced ferroptosis.

**FIGURE 9:**
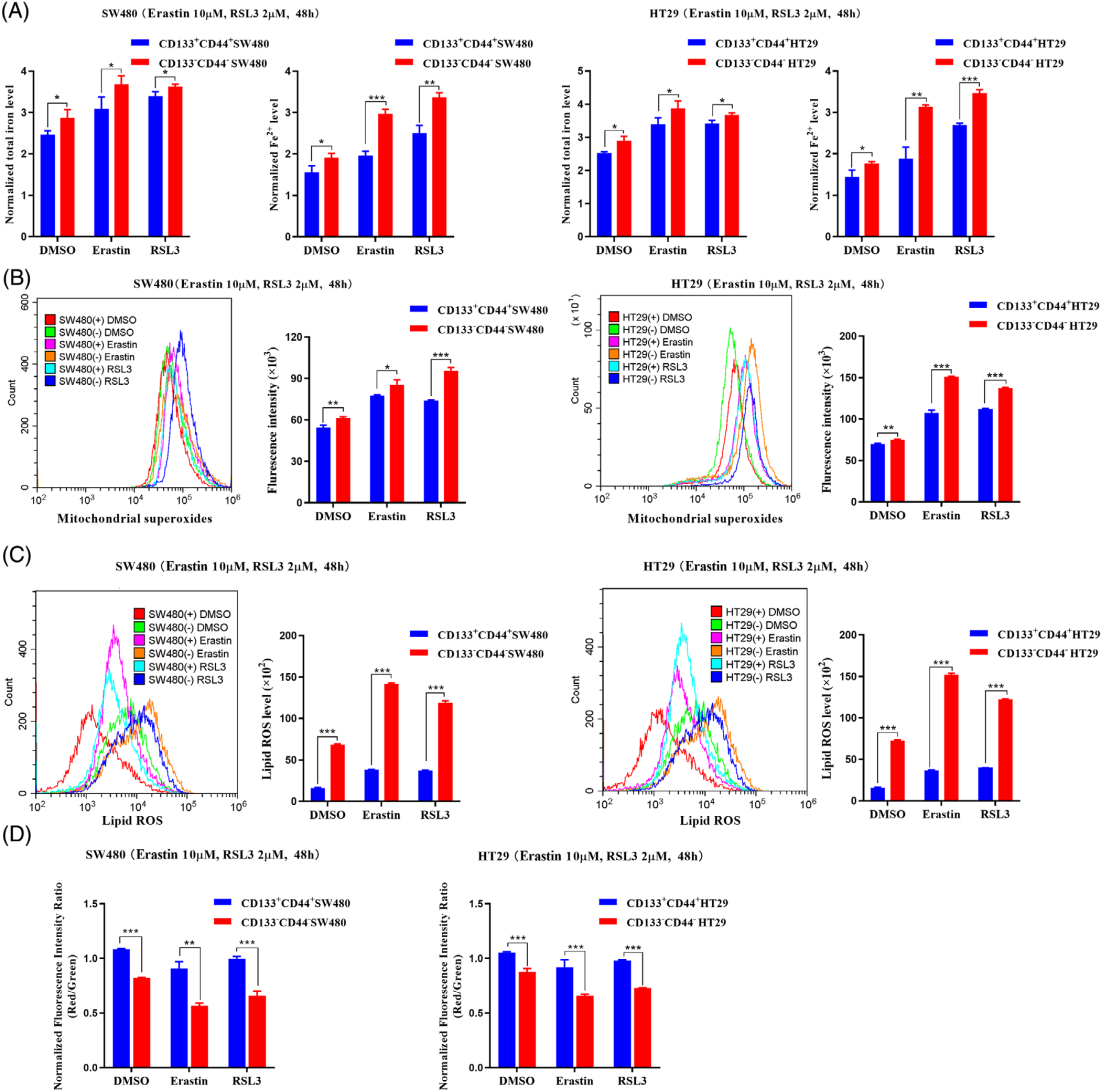
Colon cancer stem cells suppress ferroptosis. (A) Levels of intracellular total iron and Fe^2+^ were analysed in CD44+CD133+ and CD44−CD133− ‐SW480 and ‐HT29 cells subjected to Erastin (10 μM) and RSL3 (2 μM) for 48 h (*n* = 3). (B) Flow cytometry analysis the intracellular mitochondrial superoxide level in CD44+CD133+ and CD44−CD133− ‐SW480 and ‐HT29 cells subjected to Erastin (10 μM) and RSL3 (2 μM) for 48 h (*n* = 3). (C) Flow cytometry analysis the level of intracellular lipid ROS in CD44+CD133+ and CD44‐CD133− ‐SW480 and ‐HT29 cells subjected to Erastin (10 μM) and RSL3 (2 μM) for 48 h (*n* = 3). (D) Levels of mitochondrial membrane potential were analysed in CD44+CD133+ and CD44−CD133− ‐SW480 and ‐HT29 cells subjected to Erastin (10 μM) and RSL3 (2 μM) for 48 h (*n* = 3). Data are shown as the mean ± SD. *p < .05, **p < .01 and ***p < .001 compared with control. SW480(+), CD44+CD133+− SW480; SW480(−), CD44−CD133−SW480; HT29(+), CD44+CD133+−HT29; HT29(−), CD44−CD133− HT29(+)

We then explored the role of colon CSCs in the process of Erastin‐ or RSL3‐induced ferroptosis in CD44^+^CD133^+^ and CD44^−^CD133^−^ ‐SW480 and ‐HT29 cells, which was sorted by double stained with anti‐CD44‐PE and anti‐CD133‐FITC (Figure 3(B)). Compared with CD133^−^CD44^−^ colon cancer cells, the intracellular concentrations of total iron and Fe^2+^ (Figure 9(A)), mitochondrial superoxide (Figure 9(B)) and lipid ROS (Figure 9(C)) were substantially higher than those in CD133^−^CD44^−^ cells, whereas the mitochondrial membrane potential was significantly lower (Figure 8(D)), indicating that CD44^+^CD133^+^ colon CSCs blocked the process of ferroptotic cell death. Overall, these data suggested that LINC01606–Wnt/β‐catenin axis signalling might promote stemness in colon cancer cells by blocking the process of ferroptosis.

### 3.8 LINC01606 protects cells from ferroptosis by increasing the formation of MUFAs

**FIGURE 10:**
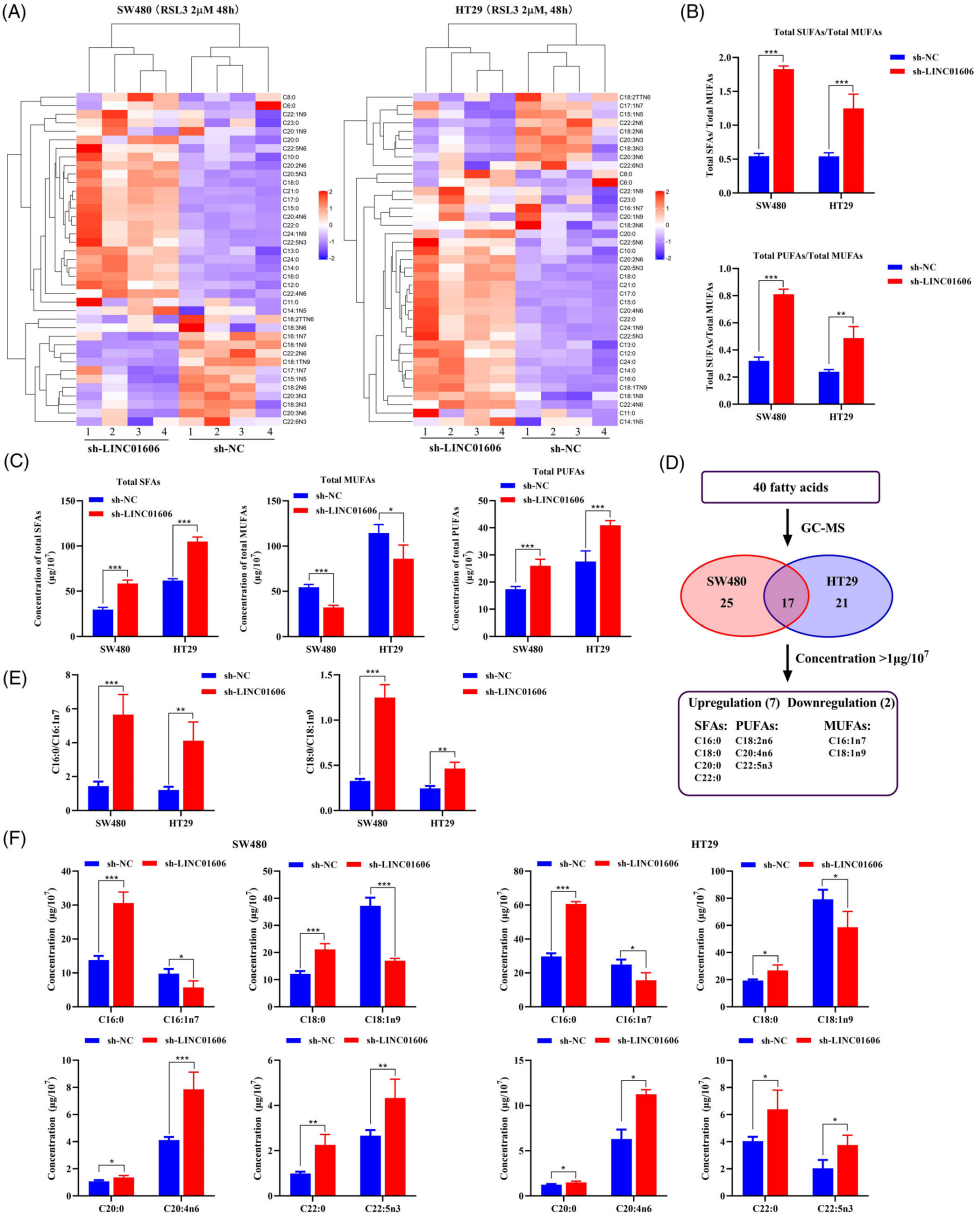
Alterations in fatty acids concentration profiles in colon cancer cells. (A) The result from Hierarchical clustering heatmap shows distinguishable fatty acids concentration profiling among cells. Fatty acids concentration levels are indicated as follows: ‘Red’ indicates high relative concentration; and ‘Blue’ indicates low relative concentration (*n* = 4). (B) The ratio between total concentration of SFAs and total MUFAs or PUFAs in LINC01606 knockdown or control SW480 and HT29 cells subjected to RSL3 (2 μM) for 48 h. (C) The concentration of total SFAs, MUFAs and PUFAs in in LINC01606 knockdown or control SW480 and HT29 cells subjected to RSL3 (2 μM) for 48 h. (D) The concentration of 40 fatty acids were detected by GCMS in LINC01606 knockdown or control SW480 and HT29 cells subjected to RSL3 (2 μM) for 48 h. (E) The ratio between concentration of palmitic acid (C16:0) or stearic acid (C18:0) and palmitoleic acid (C16:1n7) or oleic acid (C18:1n9) in LINC01606 knockdown or control SW480 and HT29 cells subjected to RSL3 (2 μM) for 48 h. (F) The concentration of palmitic acid (PAL, 16:0), stearic acid (C18:0), palmitoleic acid (C16:1n7), oleic acid (C18:1n9), arachic acid (C20:0), arachidonic acid (C20:4n6), behenic acid (C22:0) and docosapentaenoic acid (C22:5n3) in LINC01606 knockdown or control SW480 and HT29 cells subjected to RSL3 (2 μM) for 48 h. Data are shown as the mean ± SD. *p < .05, **p < .01 and ***p < .001 compared with control

Ferroptosis is tightly related to the FA balance and driven by peroxidation of PUFAs, and the formation of oxidised membrane PUFAs is a hallmark of ferroptosis. Therefore, we questioned whether LINC01606 prevented ferroptosis through preventing the formation of lipid species. To clarify and distinguish the composition of lipids, we first probed changes in lipid composition induced by LINC01606 blockade using GC–MS. As expected, the effects of LINC01606 blockade on the fatty acid composition using lipidomic analysis compared the relative levels of approximately 40 individual lipid species, including SFAs, MUFAs and PUFAs, in SW480 and HT29 cells that had been treated with the inducer of ferroptosis RSL3 (Figure 10(A)). We further noted that the ratios of SFAs/MUFAs and PFAs/MUFAs were significantly increased across all lipid classes with LINC01606 knockdown (Figure 10(B)), and the concentrations of total SFAs and PUFAs were significantly increased, whereas the concentration of total MUFAs was significantly decreased in SW480 and HT29 cells (Figure 10(C)). We calculated the differential mass signal intensities of lipid species between SW480 and HT29 cells to generate differential lipidomic profiles and identified 25 and 21 fatty acids with significant changes in SW480 and HT29 cells, respectively (Tables S3 and S4). Combining the two cell lines, 17 lipid species showed significant changes in both cell lines, but nine fatty acids (two downregulated and seven upregulated) displaying the most significant changes (>1 μg/10^7^) were identified upon LINC01606 knockdown (Figure 10(D)).

Blocking LINC01606 increased the concentrations of SFAs containing palmitic acid (C16:0), stearic acid (C18:0), arachidic acid (C20:0) and behenic acid (C22:0) and PUFAs containing linoleic acid (C18:2n6), arachidonic acid (C20:4n6) and docosapentaenoic acid (C22:5n3) but decreased the concentrations of MUFAs containing palmitoleic acid (C16:1n7) and oleic acid (C18:1n9) (Figure 10(F)). Among lipids that were dysregulated upon LINC01606 knockdown, the most notable were palmitic acid, stearic acid, palmitoleic acid and oleic acid. SCD1 is the rate‐limiting enzyme catalysing the desaturation of SFAs, principally stearic acid and palmitic acid, to their counterparts, oleic acid and palmitoleic acid. We observed that the ratio of palmitic acid/palmitoleic acid (C16:0/C16:1n7) and stearic acid/oleic acid (C18:0/C18:1n9) significantly increased (Figure 10(E)), further indicating that LINC01606 blockade induced ferroptosis, consistent with SCD1 inhibition‐induced ferroptosis by preventing the formation of SFAs and PUFAs or promoting the formation of MUFAs.

Ferroptosis has been linked to the oxidation of PUFAs, particularly phosphatidylethanolamine (PE) containing linoleic acid, arachidonic acid and adrenic acid, which are easily attacked by ROS, while MUFAs are much less reactive.^40^, ^41^ These findings are consistent with our observation that LINC01606 depletion triggered ferroptosis by increasing the synthesis of linoleic acid, arachidonic acid and docosapentaenoic acid. However, we noted no increase in adrenic acid. Collectively, these results suggested a functional role for LINC01606 in ferroptosis by altering the ratio of saturated to unsaturated fatty acids, and ferroptosis was triggered by depletion of PUFA oxidation.

### 3.9 Identification of LINC01606 transcriptional regulation by the transcription factor TFE3

**FIGURE 11:**
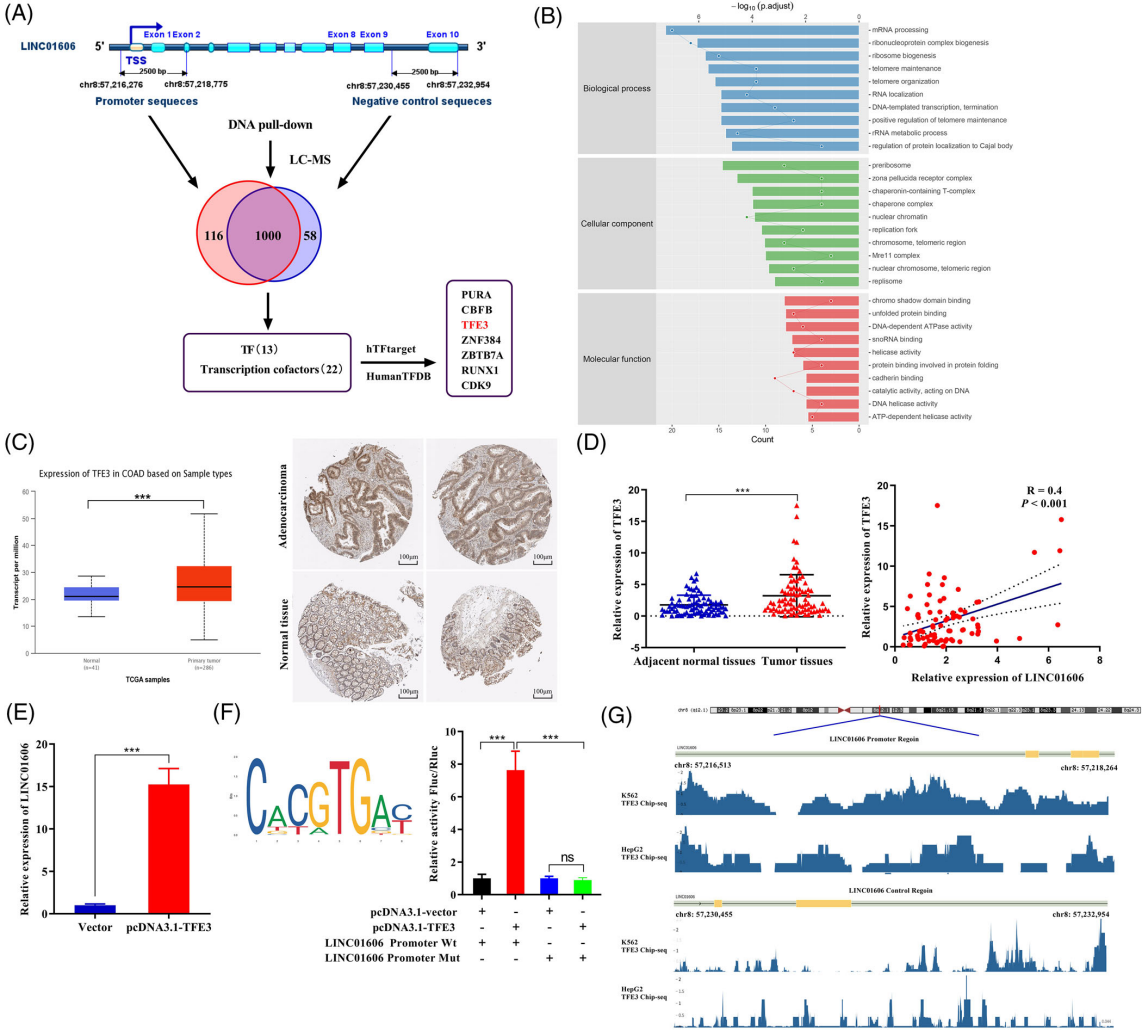
Identification of transcription factor TFE3 binding to the LINC01606 promoter. (A) Transcription related‐nucleoprotein binding to the LINC01606 promoter was detected by DNA pull‐down and LC–MS in SW480 cells. (B) Gene Ontology enrichment analysis of differentially expressed of nucleoprotein binding to LINC01606 promoter region. (C) Protein and mRNA expression level of TFE3 in TCGA colon rectal cancer tissues and normal tissues. (D) TFE3 expression levels were assessed in human colon cancer tissues compared with the paired adjacent normal tissues (*p* < .001, *n* = 83). Association between TFE3 levels and LINC01606 levels in 83 colon cancer patients. (E) Relative expression of LINC01606 in SW480 cells transfected with TFE3 or control plasmid (*n* = 3). Expression levels were normalised to GAPDH levels. (F) JASPAR was used to predict sites of putative TFE3 binding motif in the LINC01606 promoter region. Luciferase reporting gene assay was performed to analyse whether TFE3 could bind to LINC01606 promoter region (*n* = 3). (G) ENCODE database predicted the TFE3 Chip‐seq signal values on LINC01606 promoter region in A562 and HepG2 cells. Data are shown as the mean ± SD. *p < .05, **p < .01 and ***p < .001 compared with control. TSS, transcription start site; TF, transcription factor

To explore the molecular mechanism responsible for the positive feedback regulatory loop between LINC01606 and Wnt/β‐catenin signalling, we first focused on the classic TCF/LEF transcription factor (TF) family, LEF1 and TCF7, and we used siRNA to deplete these factors. We found that LINC01606 expression significantly decreased after depletion of these factors in SW480 and HT29 cells (Figure S3). We then performed DNA pull‐down/LC–MS experiments to explore TF binding to the LINC01606 promoter region involved in transcription in SW480 cells. Beginning from the transcription start site (TSS), the DNA sequence 2 kilobases (kb) upstream and 0.5 kb downstream was defined as was LINC01606 promoter probe, whereas the DNA sequence of the last exon and part of the intron was defined as the NC probe (Table S5). We identified 1116 and 1058 nucleoproteins that had significant changes in the promoter region and NC region, respectively (Figure 11(A)). In this differential protein profile, we identified 116 nucleoproteins that bound to the LINC01606 promoter region, excluding nonspecific binding in the NC, including 13 TFs and 22 transcription cofactors. According to the Gene Ontology analysis, the main functions of these differential nucleoproteins were focused on DNA edition and transcription (Figure 11(B)). Combined with potential LINC001606 promoter region binding TFs predicted in the HumanTFDB and hTFtarget databases, we screened 7 TFs and cofactors in these differential protein profiles and finally focused on TFE3, a microphthalmia family of TFs (MiTF).

Based on the above findings, we first used the TCGA database to identify TFE3 mRNA and protein expression levels in colon cancer samples and normal tissue samples. These data indicated that TFE3 was markedly upregulated in cancer tissues compared with normal tissues (Figure 11(C)). Furthermore, we detected TFE3 mRNA level in 83 pairs of colon cancer samples and adjacent normal tissue samples. Consistent with the TCGA data, TFE3 expression was upregulated in colon cancer tissues compared with adjacent normal tissues (Figure 11(D)). Subsequently, we found a strong correlation between LINC01606 and TFE3 expression in 83 colon cancer patients (Figure 11(D)). Next, we found that LINC01606 expression was significantly increased after overexpression of TFE3 by pcDNA3.1–TFE3 plasmids (Figure 11(E)). JASPAR was further used to predict potential TFE3 binding sites that could bind to the promoter regions of LINC01606. Although there are many TFE3 TF binding sites in the LINC01606 promoter, these binding sites need further verification. To test the functional results of TFE3 binding to LINC01606 promoter region, the seven predicted TFE3 binding site of LINC01606 promoter region WT and a mutated TFE3 binding site of LINC01606 promoter region mutant type were cloned into luciferase reporter plasmids (Figure S6). It was observed that TFE3 significantly increased luciferase activity in the LINC01606 promoter region WT vector but not in the LINC01606 promoter region mutant vector following transfection of pcDNA3.1–TFE3, pcDNA3.1, LINC01606 promoter–WT or LINC01606 promoter–MUT vector (Figure 11(F)) in SW480 cells. It is noteworthy that the TFE3 Chip‐seq signal values observed an enrichment of TFE3 peaks in the LINC01606 promoter region in the ENCODE database (Figure 11(G)). Therefore, we confirmed that TFE3 was functionally involved in the LINC01606 promoter region and promoted LINC01606 transcription; however, further analyses are required.

## 4 DISCUSSION

**FIGURE 12:**
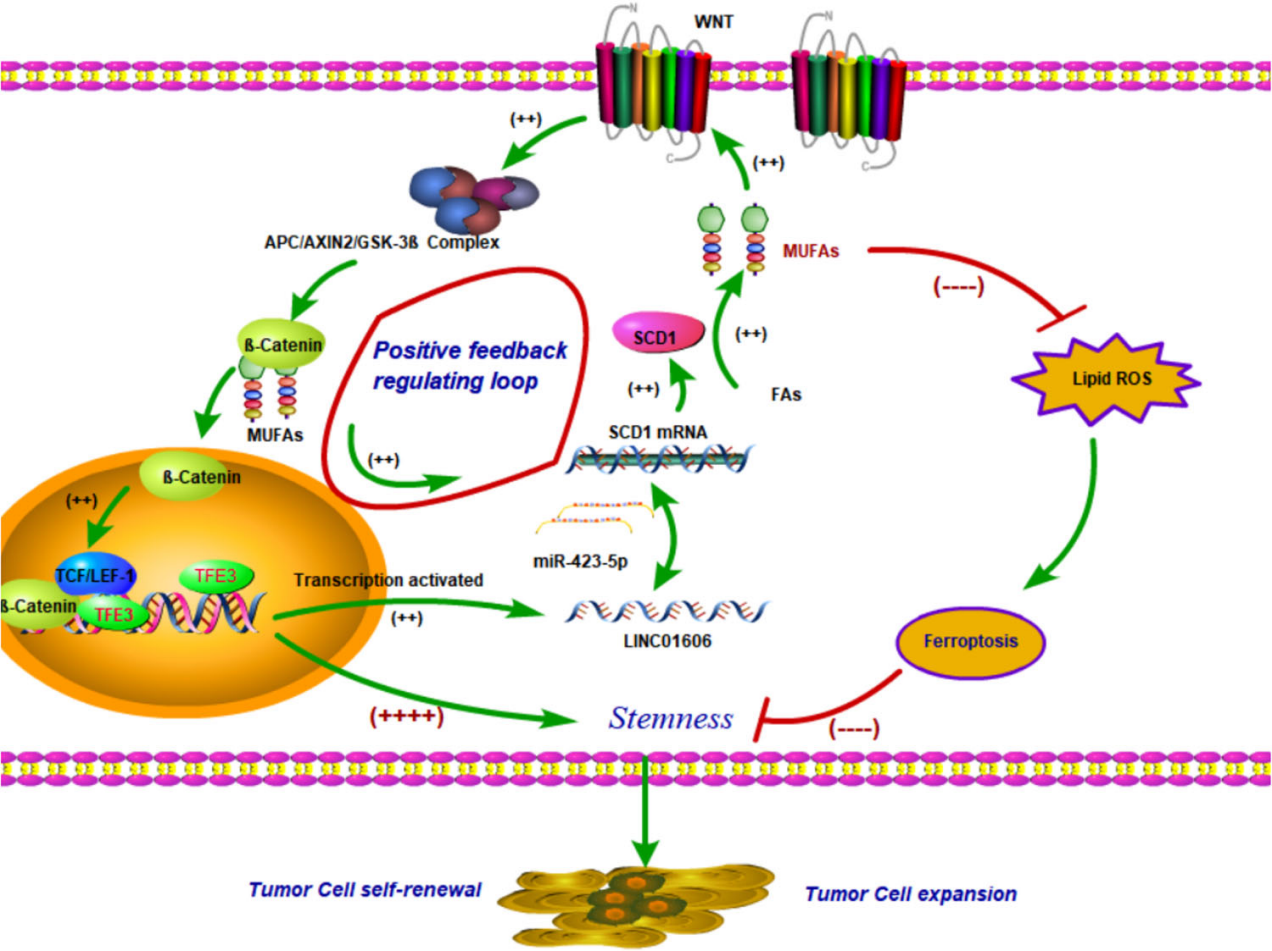
A schematic model of LINC01606–SCD1–Wnt/β‐catenin signalling circuit in colon cancer cells. LINC01606 activates Wnt/β‐catenin signalling by SCD1, promoting cancer cell growth and stemness and inhibiting ferroptosis. Simultaneously, activating Wnt/β‐catenin signalling in turn enhances LINC01606 expression by TFE3, forming a LINC01606–SCD1–Wnt/β‐catenin signalling circuit

Tumour progression accounts for the majority of cancer‐related deaths, and few good therapeutic options are available after progression. The discovery of ferroptosis, a mechanism of cell death to which colon cancer cells are susceptible,^42^ opens the window to new therapies for this disease. In the current study, we showed that LINC01606 acted as an oncogene and predicted poor survival in colon cancer, that genetic blockade of LINC01606 inhibited proliferation and stemness and induced apoptosis and ferroptosis, and that pharmacologic or genetic blockade of Wnt/β‐catenin signalling enhanced sensitivity to ferroptosis inducers in a model of cCSCs and in colon cancer cell lines. LINC01606 promoted SCD1 expression by interacting with miR‐423‐5p and subsequently activated Wnt/β‐catenin signalling by controlling the synthesis of intracellular MUFAs, whereas activation of Wnt/β‐catenin signalling enhanced LINC01606 expression by TF TFE3, which could continuously increase the synthesis of MUFAs and neutralise the increase in lipid ROS under oxidative stress caused by ferroptosis inducers, which are necessary to maintain the stemness and ferroptosis resistance of cancer cells (Figure 12).

In our previous study, we reported that LINC01606 facilitated Wnt3a expression by miR‐423‐5p to activate Wnt/β‐catenin signalling.^31^ To test this hypothesis, we first demonstrated that LINC01606 was a cytoplasmic lncRNA. Then, we blocked LINC01606 or miR‐423‐5p, and the LINC01606 or miR‐423‐5p overexpression caused corresponding changes in Wnt3a expression. There were no functional binding sites between miR‐423‐5p and the Wnt3a 3′UTR. We wondered how LINC01606 and miR‐423‐5p regulated Wnt/β‐catenin signalling in colon cancer and why Wnt3a expression was changed, consistent with our predictions. Therefore, we hypothesised that LINC01606 might regulate SCD1, a Wnt3a upstream molecule, to activate Wnt/β‐catenin signalling. According to verification of the above series of experiments, we identified that miR‐423‐5p was directly bound to the SCD1 3′UTR, and the sequence of the binding site was the same as that of LINC01606 (Figure 4). We further demonstrated that LINC01606 activated this signalling by increasing β‐catenin‐mediated TCF/LEF transcriptional activity (Figure 7). These results indicated that LINC01606 activated Wnt/β‐catenin signalling by increasing SCD1 but not Wnt3a levels.

Interestingly, we found that pharmacologic activation of Wnt/β‐catenin signalling enhanced LINC01606 and SCD1 expression, inversely pharmacologic blockade of Wnt/β‐catenin signalling suppressed LINC01606 and SCD1 expression. Therefore, we audaciously hypothesised that LINC01606 formed a positive feed‐forward loop with SCD1‐Wnt/β‐catenin signalling. To validate this hypothesis, we interfered with the expression of the classic TCF/LEF TF families LEF1 and TCF7, both of which caused a downregulation of LINC01606 and SCD1. These results were puzzling. Next, to illustrate this regulatory relationship, we used a promoter pull‐down assay and found that TF TFE3 might bind to the LINC01606 promoter. TFE3, belonging to the MiT family of helix‐loop‐helix leucine zipper TFs, is known to participate in the biogenesis of lysosomes and autophagosomes and the clearance of cellular debris via activation of CLEAR elements.^43^ Accumulating evidence has demonstrated that TFE3 plays a vital role in Wnt/β‐catenin signalling. The functional cooperation of TFE3 and LEF1 can enhance WNT pathway activation.^44^, ^45^ Additionally, TFE3 has been reported to enhance Wnt signalling by increasing GSK3 sequestration,^46^ and TFE3 gives rise to positive feedback loop regulation of Wnt signalling by GSK3.^47^ We found that TFE3 was markedly upregulated in colon cancer and had a strong correlation with LINC01606. And overexpression of TFE3 could increase LINC01606 expression, demonstrating that LINC01606 expression was positively regulated by TFE3. According to further analysis, many TFE3 binding sites were present in the LINC01606 promoter region. Furthermore, we identified that TFE3 was directly bound to the LINC01606 promoter region (Figure 11). These data suggested that TFE3 could exert a promoting effect on LINC01606 expression in colon cells and form a positive feedback loop of LINC01606–SCD1–Wnt/β‐catenin–TFE3 involved in cancer progression, but further verification is needed.

Notably, multiple lines of evidence have recently highlighted the dual role of lncRNAs in ferroptotic cell death. LINC00336 inhibits ferroptosis through decreasing the concentrations of iron, mitochondrial superoxide and lipid peroxidation and increasing mitochondrial membrane potential.^28^ In contrast, the lncRNAs MT1DP,^29^ LINC00618,^30^ PVT1,^48^ ZFAS1,^49^ GABPB1‐AS1^50^ and P53RRA^51^ accelerate ferroptosis by increasing lipid peroxidation, iron deposition and mitochondrial superoxide. Consistently, we demonstrated herein that LINC01606 decreased the concentration of iron, mitochondrial superoxide and lipid peroxidation and increased mitochondrial membrane potential, in agreement with its role in ferroptosis, indicating that LINC01606 acted as a ferroptosis suppressor. Furthermore, pharmacologically activated or blockaded Wnt/β‐catenin signalling could also inhibit or accelerate ferroptosis, respectively, and increased LINC01606 could attenuate blocked Wnt/β‐catenin signalling‐induced ferroptosis, indicating that activation of Wnt/β‐catenin signalling also acted as a ferroptosis suppressor. We also explored the relationship between colon CSCs and ferroptosis and found that colon CSCs had low concentrations of iron, lipid ROS and mitochondrial superoxide and an increased mitochondrial membrane potential, indicating that colon CSCs possessed the ability to resist ferroptosis. Notably, LINC01606 and SCD1 were upregulated in colon CSCs, and LINC01606 enhanced the stemness of cancer cells, indicating that LINC01606 might confer ferroptosis resistance ability to colon CSCs.

Lipids are crucial regulators of cell death, acting both as initiators and facilitators.^52^ Lipid oxidation is likely central to the process of ferroptotic cell death. Therefore, we explored the particular types of lipid species that participated in the blockade of LINC01606‐induced ferroptosis. We observed that interference with lipid homeostasis through blockade of LINC01606 induced multiple changes in cellular lipid content (Figure 10). SCD1 is a LINC01606 target gene that converts palmitic acid and stearic acid to palmitoleate and oleate, respectively. As expected, we found that blockade of LINC01606 induced high levels of palmitic acid and stearic acid and low levels of palmitoleate and oleate in ferroptosis, further supporting SCD1 as a target gene for LINC01606. Consistent with our findings, Tesfay et al.^53^ reported that overexpression of SCD1 or exogenous administration of palmitoleic acid or oleate products protected cells from ferroptotic cell death. Zhao et al.^39^ also reported that SCD1 protected cells from ferroptosis by shifts in MUFAs and PUFAs, particularly by increasing levels of PE. These results indicate that LINC01606 protects against ferroptotic cell death by increasing levels of the MUFAs palmitoleate and oleate. Conversely, ferroptotic cell death has been linked to the oxidation of PUFAs, linoleic acid or particularly PE containing arachidonic acid and adrenic acid. However, we observed no increase in adrenic acid but an increase in docosapentaenoic acid.^41^, ^42^ In fact, the converse appeared to be true: except for common PUFAs, which are the most susceptible lipids to peroxidation, other uncommon PUFAs are also susceptible to redox oxidation products, such as PUFA‐OOH, during the course of ferroptosis, reflecting the oxidative destruction of these species during ferroptosis.^54^ Additionally, docosapentaenoic acid has been found to induce apoptosis and reduce cell proliferation of colorectal cancer cells, which have anticarcinogenic effects,^55^ further indicating that blockade of LINC01606 inhibits tumour growth in part by increasing docosapentaenoic acid levels, the anticarcinogenic effects of which may induce ferroptotic cell death through its own oxidation. These findings strongly indicate that PUFAs play a vital role in the blockade of LINC01606‐triggered ferroptosis.

In summary, this work illustrates that LINC01606 acts as an oncogene to promote cancer cell stemness and inhibit ferroptosis in the positive feed‐forward loop between LINC01606 and Wnt/β‐catenin signalling. These findings indicate that LINC01606 is a crucial therapeutic target for cancer progression and may provide a promising option for colon cancer treatment.

## CONFLICT OF INTEREST

The authors declare no conflict of interest.

## Supporting information

Supporting Information
