# Supplementary material for: Long noncoding RNA LINC01606 protects colon cancer cells from ferroptotic cell death and promotes stemness by SCD1–Wnt/β‐catenin–TFE3 feedback loop signalling
Source: Clin Transl Med. 2022 Apr 29;12(4):e752. doi: 10.1002/ctm2.752 (PMC9052012; doi:10.1002/ctm2.752)
Supplement: Supplementary file 1 — Supporting Information [file CTM2-12-e752-s001.pdf]

Additional files

Supplemental Figures

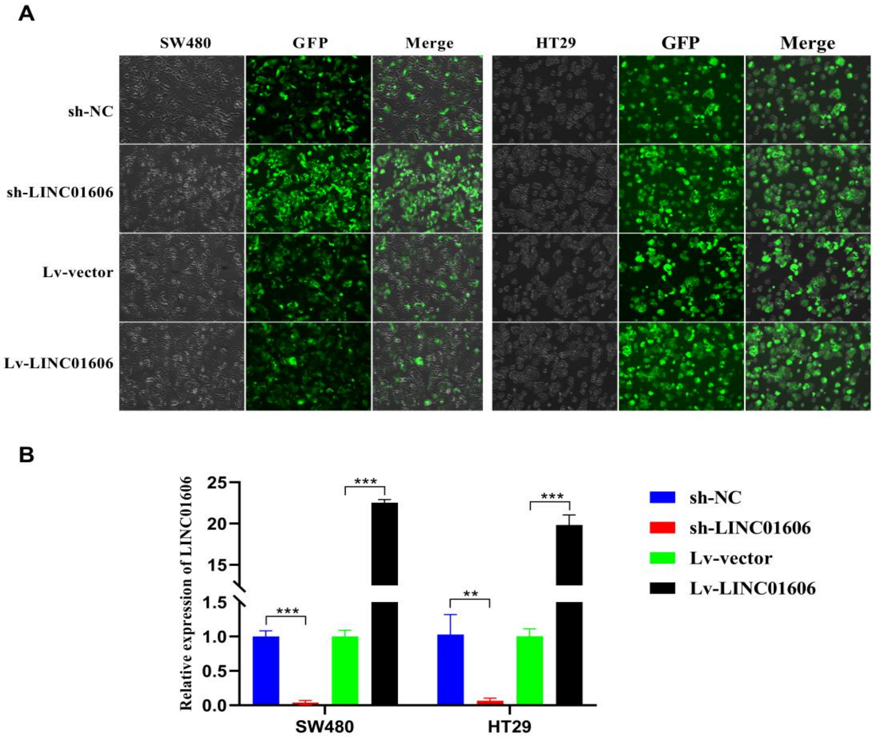

Figure S1. The efficiency of knockdown and overexpression of LINC01606 in colon cancer cells. (A) SW480 and HT29 cells were transfected with shRNA-LINC01606-expressing vector (sh-LINC01606) and shRNA-control vector (sh-NC) or lentivirus-LINC01606-expressing vector (Lv-LINC011606) and lentivirus-control vector (Lv-vector), cells were observed under a fluorescence microscope. **(B)** The efficiency of stable knockdown and overexpression of LINC016016 was detected by qRT-PCR (n = 3). Data are shown as the mean ± SD. **$p < 0.01$ and ***$p < 0.001$ compared with control.

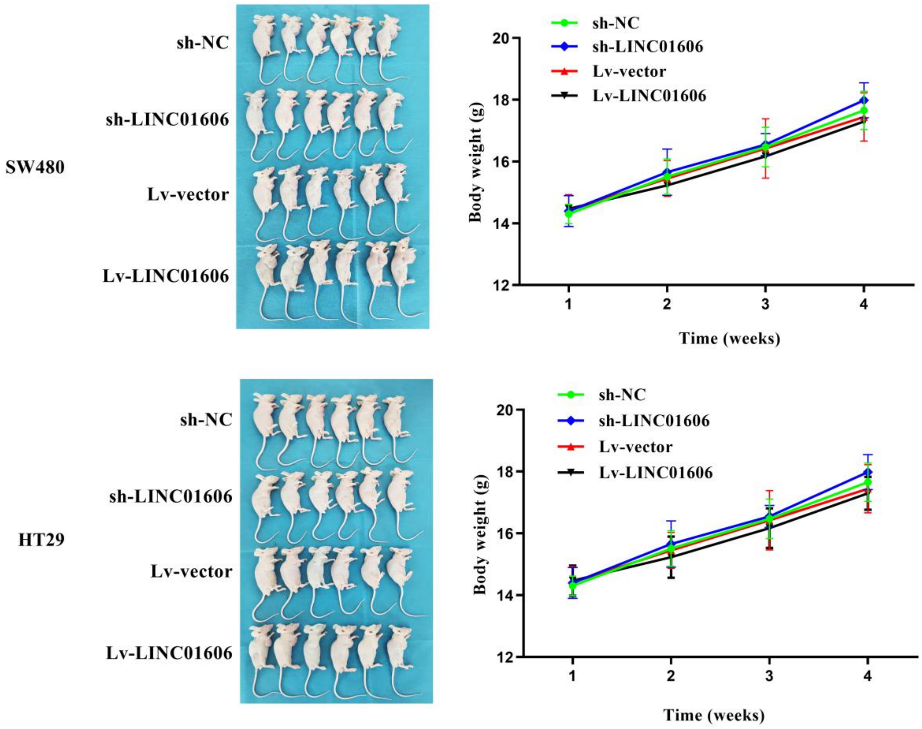

Figure S2. The whole-body weight of xenografts mice. Representative images of tumors in xenograft mouse models bearing SW480 and HT29 cells transfected with sh-LINC01606-expressing vector, LINC01606-expressing vector and each control vector. The whole body weight was measured every week when mice were sacrificed ($p > 0.05$, n = 6). Data are shown as the mean ± SD. $p > 0.05$ compared with control.

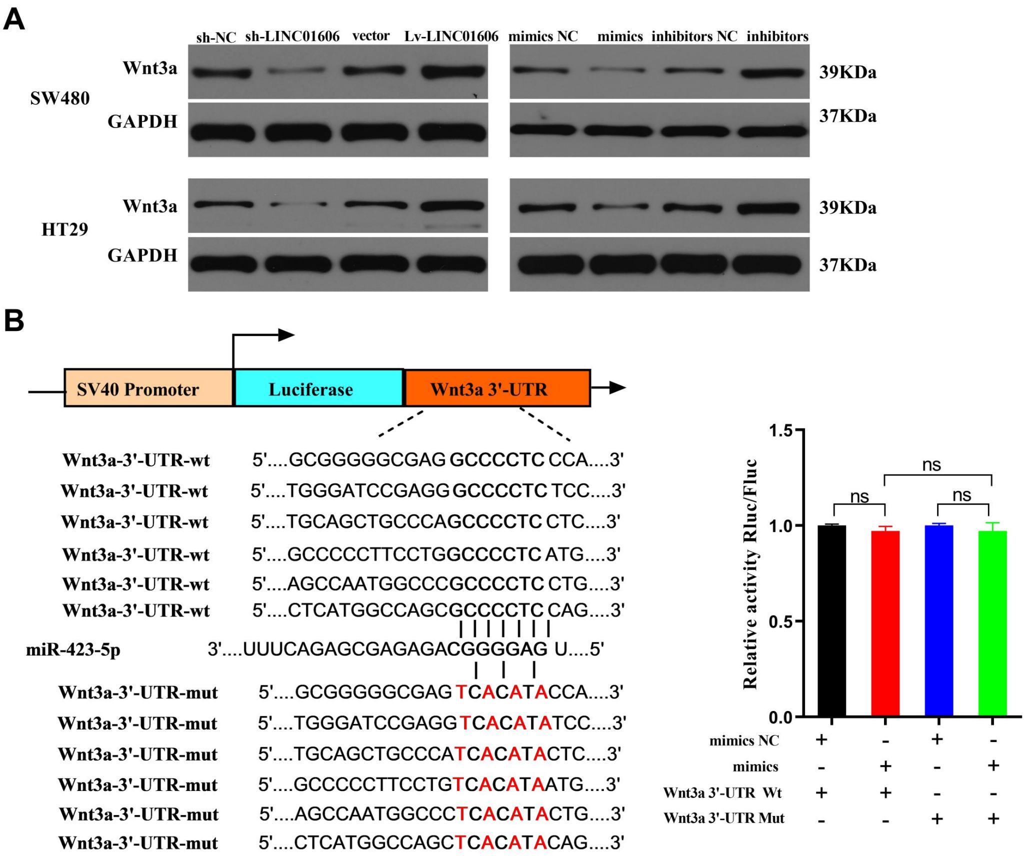

Figure S3. (A) Protein expression level of Wnt3a in SW480 and HT29 cells after transfection with sh-LINC01606-expressing vector or LINC01606-expressing vector and miR-423-5p mimics or inhibitors (n = 3). **(B)** The binding sites of miR-423-5p on Wnt3a 3'UTR. Co-transfection of miR-423-5p and Wnt3a 3'UTR-Wt or Wnt3a 3'UTR-Mut did not change the luciferase activity in HEK293T cells (n = 3). Data are shown as the mean ± SD. $p > 0.05$ compared with control. ns: no significant.

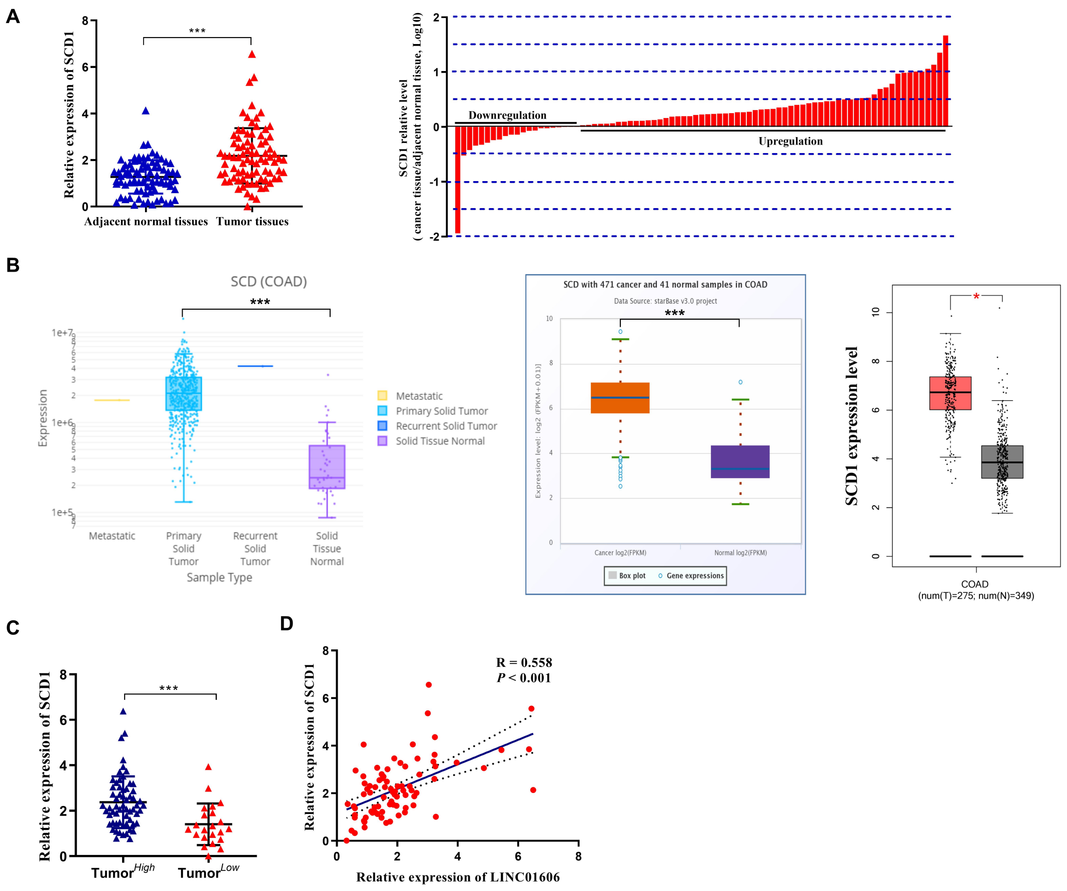

Figure S4. Expression level of SCD1 in colon cancer. (A) SCD1 expression is higher in colon cancer tissues compared with the paired adjacent normal tissues ($p < 0.001$, n = 83). Colon cancer tissues express significantly higher levels of SCD1 than paired adjacent normal tissues in the majority of patients (61/83, 77.11%). Expression levels were normalized to GAPDH levels. **(B)** SCD1 expression is higher in colon cancer tissues compared with the normal tissues in TCGA database from DriverDBv3, starBase and GEPIA. **(C)** SCD1 expression level is higher in LINC01606 upregulated tumor tissues (n = 61) than those in LINC01606 downregulated tumor tissues (n = 22). Expression levels were normalized to GAPDH levels. **(D)** Association between SCD1 levels and LINC01606 levels in 83 colon cancer patients. Data are shown as the mean ± SEM. *$p < 0.05$, **$p < 0.01$ and ***$p < 0.001$ compared with control.

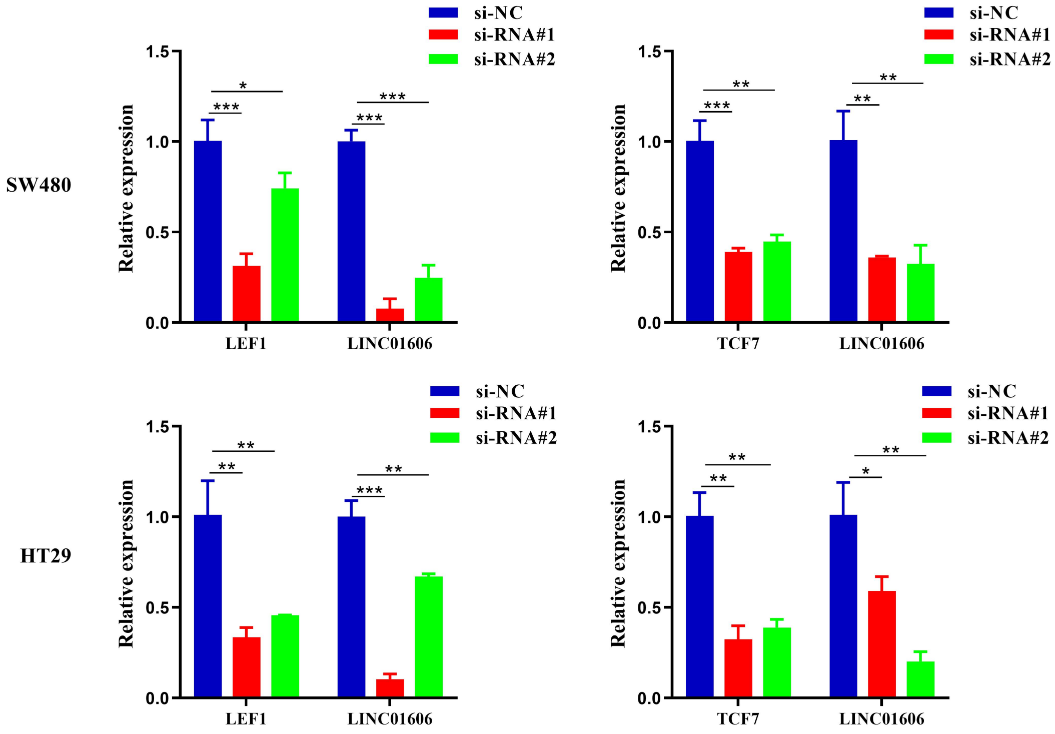

Figure S5. Expression level of LINC01606 in colon cancer. The expression level of LINC01606 in SW480 and HT29 cells after transfected with siRNA of LEF1 and TCF7. Expression levels were normalized to GAPDH levels (n = 3). Data are shown as the mean ± SD. *$p < 0.05$, **$p < 0.01$ and ***$p < 0.001$ compared with control.

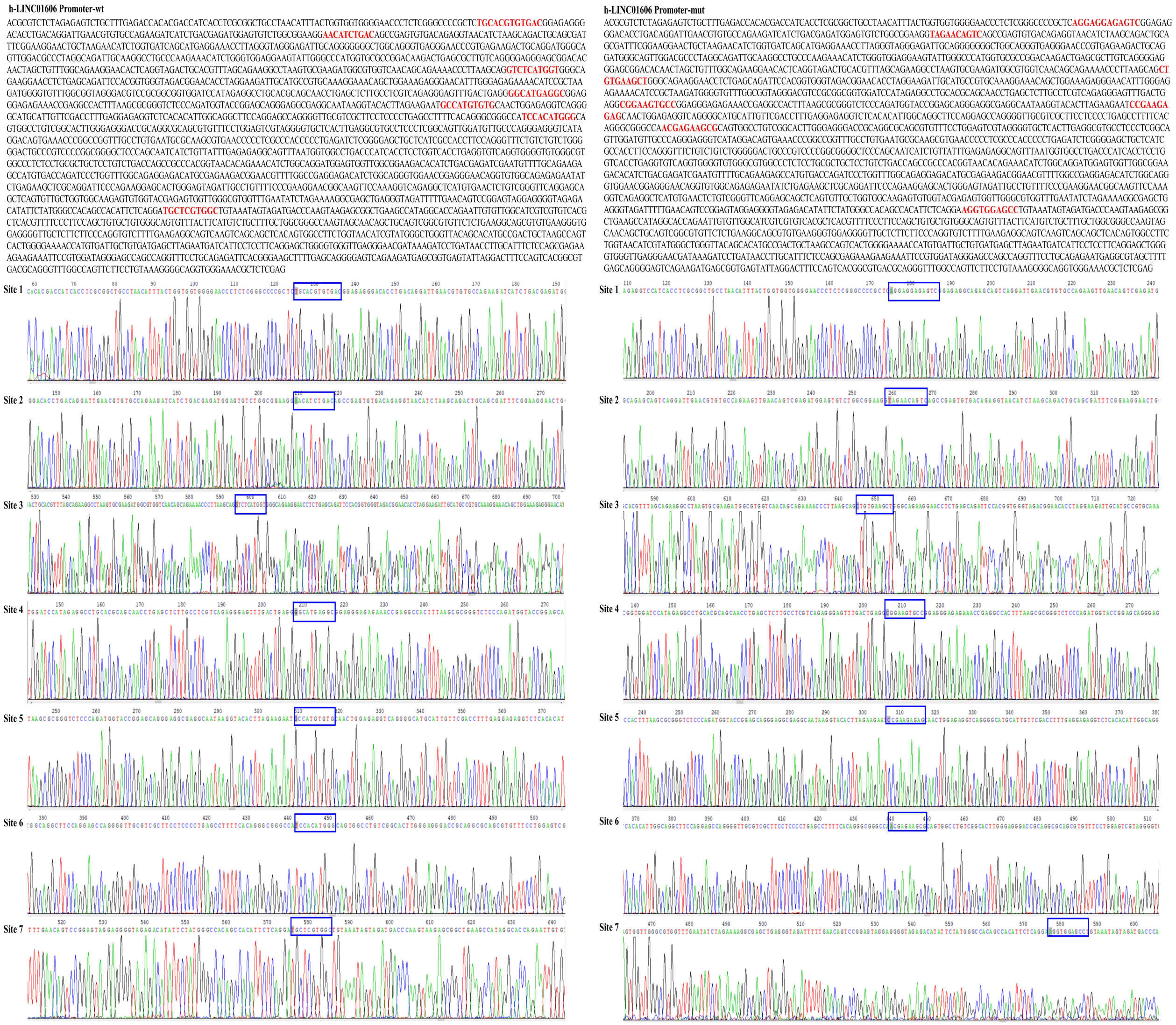

Figure S6. The sequencing results for the designed wild type and mutant type of TFE3 binding sits for LINC01606 promoter. Binding sites were marked in red.

Supplemental Tables

Table S1. shRNA, siRNA and miRNA sponge and probe target sequences

Gene name	Source	Target sequences
sh-LINC01606	Genechem	Sense:5’-GATCCGCGAGAAAGAAGAAATTCCGTGGATATTCAAGAGATATCCACGGAATTTCTTCTTTCTCGTTTTTTG-3’<br>Antisense:5’-AATTCAAAAAACGAGAAAGAAGAAATTCCGTGGATATCTCTTGAATATCCACGGAATTTCTTCTTTCTCGCG-3’
si-LEF1 #1	Ruibio	5’-GGTACATAATGATGCCAAA-3’
si-LEF1 #2	Ruibio	5’-GCCTCAGCATGAACAGAGA-3’
si-TCF7 #1	Ruibio	5’-CCAACTCTCTCTCTACGAA-3’
si-TCF7 #2	Ruibio	5’-GAGGGAAAAGCACCAAGAA-3’
miR-423 sponge	Sangon	5’-AAAGUCUCGCUCUCUGCCCCUCA-3’
Bio-miR-423-wt probe	Sangon	5’-UGAGGGGCAGAGAGCGAGACUUU-3’
Bio-miR-423-mut probe	Sangon	5’-AGUGCGCCUGUGUGGGUGUCUUU-3’

Table S2. The primer sequences used for real-time quantitative PCR

Gene name		The primer sequences	Amplification length (bp)
LINC01606	Forward	5'-GCTGGACATTTCTCCCTTCA-3'	118 bp
	Reverse	5'-GAGTCCTCTCGCTTCCTCCT-3'	
SCD1	Forward	5'-CACCACATTCTTCATTGATTGCA-3'	75 bp
	Reverse	5'-ATGGCGGCCTTGGAGACT-3'	
Wnt3a	Forward	5'-CTGTTGGGCCACAGTATTCC-3'	113 bp
	Reverse	5'-GGGCATGATCTCCACGTAGT-3'	
β-catenin	Forward	5'-CATCTACACAGTTTGATGCTGCT-3'	150 bp
	Reverse	5'-GCAGTTTTGTCAGTTCAGGGA-3'	
c-Myc	Forward	5'-GTCAAGAGGCGAACACACAAC-3'	162 bp
	Reverse	5'-TTGGACGGACAGGATGTATGC-3'	
TCF7	Forward	5'-GACATCAGCCAGAAGCAAG-3'	142 bp
	Reverse	5'-CACCAGAACCTAGCATCAAG-3'	
LEF1	Forward	5'-AAACAGGAACATCCCCACAC-3'	106 bp
	Reverse	5'-TCAGAGGCTTCTTAATGTGAGGT-3'	
EP300	Forward	5'-TCTTTGTGATCCGCCTCAT-3'	167 bp
	Reverse	5'-GGACCACTGGGCTCTTCG-3'	
Lgr5	Forward	5'-CTCCCAGGTCTGGTGTGTTG-3'	149 bp
	Reverse	5'-GAGGTCTAGGTAGGAGGTGAAG-3'	
CD44	Forward	5'-AGTCCCTGGATCACCGA-3'	90 bp
	Reverse	5'-CCTCTTGGTTGCTGTCTCA-3'	
CD133	Forward	5'-GCATCCATCAAGTGAAACC-3'	144 bp
	Reverse	5'-ACCAGGCCATCCAAATC-3'	
Nanog	Forward	5'-CCCTGATTCTTCCACCAGT-3'	82 bp
	Reverse	5'-CGGGACCTTGTCTTCCTT-3'	
Sox2	Forward	5'-AAACGAGGGAAATGGGA-3'	148 bp
	Reverse	5'-TGTGGATGGGATTGGTG-3'	
Oct4	Forward	5'-CTTGAATCCCGAATGGAAAGGG-3'	164 bp
	Reverse	5'-GTGTATATCCCAGGGTGATCCTC-3'	
Epcam	Forward	5'-GATGGGTGAGATGCATAGGGAAC-3'	108 bp
	Reverse	5'-CGTCCCACGCACACACATT-3'	
GAPDH	Forward	5'-GCACCGTCAAGGCTGAGAAC-3'	138 bp
	Reverse	5'-TGGTGAAGACGCCAGTGGA-3'	
TFE3	Forward	5'-CCGTGTTCGTGCTGTTGGA-3'	137 bp
	Reverse	5'-CTCGTAGAAGCTGTCAGGAT-3'	
miR-423-5p	Forward	5'-GCTCTTGGAGTAGGTCATTGGGTG-3'	

Table S3. The concentration of total FAs in LINC01606 knockdown or control SW480 cells subjected to RSL3 (2 μM) for 48 h.

Fatty Acids	Unit	Fold change	P value	SW480 shRNA1	SW480 shRNA2	SW480 shRNA3	SW480 shRNA4	SW480 shNC1	SW480 shNC2	SW480 shNC3	SW480 shNC4
Methyl hexanoate	ug/10^7	1.058550015	0.867745873	0.000768634	0.000679057	0.000378216	0.000587746	0.00036766	0.000407009	0.001087634	0.000417848
Methyl octanoate	ug/10^7	1.46806773	0.141235315	0.004220579	0.005509212	0.002485554	0.003772502	0.002883277	0.002041129	0.003847548	0.002118448
Methyl decanoate	ug/10^7	1.419458843	0.012813256	0.001409128	0.001645878	0.001912688	0.001452303	0.001250649	0.001149593	0.000923735	0.001198872
Methyl undecanoate	ug/10^7	1.170866243	0.254583245	0.000484475	0.000555176	0.000677954	0.000424741	0.000542517	0.000451619	0.000406996	0.000428579
Methyl dodecanoate	ug/10^7	2.519314203	0.007996646	0.013137609	0.011407637	0.021215394	0.019303719	0.007696261	0.007343816	0.003931672	0.006854469
Methyl tridecanoate	ug/10^7	1.668974613	0.001845425	0.00187216	0.001739483	0.002130229	0.002146324	0.001204256	0.001289526	0.00086364	0.001368951
Methyl myristate	ug/10^7	2.210968588	0.00043565	1.355829718	1.497276096	1.69906235	1.866634247	0.717530532	0.864930987	0.582303309	0.738398267
Methyl myristoleate	ug/10^7	1.129209879	0.096007303	1.59437167	1.469469635	1.334349318	1.408899054	1.392299194	1.13316824	1.227470485	1.389675326
Methyl pentadecanoate	ug/10^7	2.001756939	0.000501993	0.365390232	0.373169787	0.474505036	0.352899184	0.204080554	0.18382486	0.183107266	0.211282217
Methyl cis-10-pentadecenoate	ug/10^7	0.794576391	0.141669474	0.037735674	0.039016273	0.059912463	0.051087784	0.065741168	0.067083315	0.045895262	0.057572443
Methyl palmitate	ug/10^7	2.212560678	0.0001	27.27987642	28.34996994	33.6687891	33.10371701	13.82047213	15.48895988	13.19152585	12.82062211
Methyl palmitoleate	ug/10^7	0.582781856	0.0144	4.660396753	4.446455023	8.590449546	5.120023874	8.129901742	9.580928092	9.895666437	11.54593266
Methyl heptadecanoate	ug/10^7	1.855300664	0.000132055	0.559411416	0.59801403	0.676314205	0.533907049	0.32115732	0.331914918	0.304732145	0.318348182
Methyl cis-10-heptadecenoate	ug/10^7	0.826034454	0.2186	0.794113231	0.759079009	1.312985098	0.928932931	1.178555272	1.277551312	0.945246252	1.193020012
Methyl stearate	ug/10^7	1.74202427	0.000270365	22.00992453	22.00263183	22.63438295	18.01032106	12.03102802	13.66414355	11.64916434	11.25272547
Methyl oleate	ug/10^7	0.456494679	0.0001	18.05148537	17.19364465	15.9364582	16.73602405	34.93901005	40.01875034	39.56225992	34.26071124
Methyl elaidate	ug/10^7	0.352431232	0.0003	0.215400248	0.233192847	0.267574877	0.275413808	0.775856071	0.52939695	0.712270177	0.79602365
Methyl linoleate	ug/10^7	1.424393256	0.022480144	10.0346436	7.868135621	8.218897244	6.269557368	6.069919616	5.761543165	5.251705617	5.657204527
Methyl linolelaidate	ug/10^7	1.185949758	0.079492768	0.281258153	0.308358519	0.328137436	0.374399257	0.265685165	0.311923961	0.247312277	0.264630121
Methyl linolenate	ug/10^7	0.881350673	0.167049517	0.074738496	0.07613511	0.076665488	0.085007989	0.096257581	0.092840707	0.069961466	0.095563092
Methyl γ-linolenate	ug/10^7	1.044134141	0.862871887	0.039210015	0.04092204	0.036264342	0.03783841	0.034245074	0.062141547	0.03168042	0.019648469
Methyl arachidate	ug/10^7	1.265282846	0.015238271	1.464412717	1.438107756	1.392191534	1.147712465	1.164526102	1.134198934	0.978506998	1.024118046
cis-11-Eicosenoic acid methyl ester	ug/10^7	0.993950101	0.946793055	1.118633651	1.196803365	1.276222377	1.47304808	1.232831185	1.503604783	1.127062539	1.232036439
cis-11,14-Eicosadienoic acid methyl ester	ug/10^7	2.432358117	0.000171821	0.686299345	0.620057688	0.730965747	0.584848931	0.34119779	0.309964721	0.200433685	0.226440666
cis-11,14,17-Eicosatrienoic acid methyl ester	ug/10^7	0.723272231	0.033731021	0.108875022	0.112428857	0.109408578	0.138140164	0.187069728	0.174389745	0.119426334	0.167352298
cis-8,11,14-Eicosatrienoic acid methyl ester	ug/10^7	0.880332207	0.15607273	1.072006231	1.078043282	1.192639788	1.313600715	1.419387343	1.448648875	1.099004416	1.322201288
Methyl arachidonate	ug/10^7	1.910734214	0.001113487	7.260107904	7.349075573	9.739466452	7.07268942	3.888859334	4.397329202	3.956008202	4.202444861
cis-5,8,11,14,17-Eicosapentaenoic acid methyl ester	ug/10^7	1.897715745	0.001463738	0.281084283	0.29963613	0.3343588	0.223155083	0.138073475	0.169792604	0.141712289	0.150213408
Methyl heneicosanoate	ug/10^7	1.775337462	0.000234476	0.055592403	0.0581144	0.066371406	0.051726731	0.034238927	0.032842983	0.029876238	0.03361136
Methyl behenate	ug/10^7	2.271412379	0.001650705	2.059885739	2.209236155	2.910918416	1.848889137	0.912503534	1.075803908	0.937398146	1.049323135
Methyl erucate	ug/10^7	1.175120036	0.076254129	0.596697185	0.625229464	0.674622823	0.763297028	0.561258967	0.636110865	0.496694982	0.569403104
cis-13,16-Docosadienoic acid methyl ester	ug/10^7	0.732495367	0.001629503	0.028189066	0.02976166	0.027743256	0.030306159	0.039224916	0.037828499	0.036426624	0.04488293
Methyl docosatetraenoate	ug/10^7	1.720856158	0.0023	1.388419403	1.374969231	1.048168562	1.528009716	0.746406793	0.87522186	0.804169573	0.677056645
Methyl docosapentaenoate	ug/10^7	1.626408812	0.008448373	3.617127629	4.340431782	5.496651267	3.879477549	2.658747658	3.013977451	2.500300955	2.484619232
Methyl docosapentaenoate	ug/10^7	1.215345856	0.029829986	0.289577021	0.286252927	0.357578889	0.282294257	0.263208364	0.237163867	0.258063	0.241858709
cis-4,7,10,13,16,19-Docosahexaenoic acid methyl es	ug/10^7	0.894421307	0.224122026	1.467800105	1.166723915	1.294494641	1.538585892	1.767974872	1.522776335	1.420618814	1.401637921
Methyl tricosanoate	ug/10^7	1.194439106	0.219088519	0.020517884	0.023678755	0.023251148	0.028799234	0.017116205	0.024157407	0.015397653	0.023907997
Methyl tetracosanoate	ug/10^7	1.727503032	0.000268325	0.42420469	0.428986897	0.454297743	0.512657404	0.284672634	0.26829388	0.219979689	0.280682473
Methyl cis-15-tetracosenoate	ug/10^7	1.957170876	0.003846454	4.229099843	4.634440788	6.069123515	3.968181075	2.356014759	2.973899372	2.211831917	2.115481871

Table S4. The concentration of total FAs in LINC01606 knockdown or control HT29 cells subjected to RSL3 (2 μM) for 48 h.

Name	Fatty Acids	Unit	Fold change	P value	HT29 shRNA1	HT29 shRNA2	HT29 shRNA3	HT29 shRNA4	HT29 shNC1	HT29 shNC2	HT29 shNC3	HT29 shNC4
C6:0	Methyl hexanoate	ug/10^7	1.753858509	0.092532218	0.000740913	0.001168616	0.000418538	0.000723254	0.000398619	0.000430318	0.000322436	0.000588403
C8:0	Methyl octanoate	ug/10^7	1.462507896	0.31171813	0.000945742	0.000838076	0.000165698	0.000872594	0.00041121	0.000694127	0.000545164	0.000279137
C10:0	Methyl decanoate	ug/10^7	0.928693727	0.348751994	0.003223163	0.00398529	0.003409951	0.003061023	0.003874826	0.003533236	0.003304639	0.00401705
C11:0	Methyl undecanoate	ug/10^7	1.566606301	0.030789698	0.000596063	0.001012101	0.00100841	0.001067385	0.000521493	0.000520883	0.000717738	0.000591439
C12:0	Methyl dodecanoate	ug/10^7	0.974138909	0.66578649	0.103948226	0.125075536	0.134911478	0.115857181	0.120053454	0.119204199	0.12363995	0.129632176
C13:0	Methyl tridecanoate	ug/10^7	1.11231551	0.155896636	0.007712015	0.008194197	0.008549029	0.007568751	0.008265774	0.007443352	0.006148384	0.006932875
C14:0	Methyl myristate	ug/10^7	1.208226733	0.05120904	6.184353369	6.601057501	6.794376201	6.278762087	6.51923238	5.477211038	4.526086417	4.879536892
C14:1N5	Methyl myristoleate	ug/10^7	1.003874297	0.955062888	0.655178541	0.690685648	0.6996836	0.571484781	0.73848099	0.658346255	0.604740079	0.605365217
C15:0	Methyl pentadecanoate	ug/10^7	1.687601453	0.000220135	0.561429609	0.587556492	0.609070571	0.563012652	0.417005908	0.354494818	0.290352406	0.313512668
C15:1N5	Methyl cis-10-pentadecenoate	ug/10^7	0.94333385	0.688	0.186943878	0.211837816	0.160027683	0.203848137	0.198911325	0.144984594	0.265370356	0.199204145
C16:0	Methyl palmitate	ug/10^7	2.043267689	0.002903009	60.60081774	61.90231337	58.88466689	61.43282951	28.56022153	28.04070344	32.13941258	30.09902395
C16:1N7	Methyl palmitoleate	ug/10^7	0.627628667	0.0139	14.79491723	11.59790061	22.04467568	14.0601217	29.15563443	24.76569711	22.47752423	23.17851938
C17:0	Methyl heptadecanoate	ug/10^7	1.71577508	0.004397046	1.241083046	1.584350741	1.785422415	1.233758432	0.837287571	0.80700582	0.914488177	0.8476175
C17:1N7	Methyl cis-10-heptadecenoate	ug/10^7	1.401819245	0.006	2.788742586	2.735854475	2.560301172	2.257680465	1.750357789	1.730387014	2.237771448	1.659452579
C18:0	Methyl stearate	ug/10^7	1.38320404	0.011908403	24.69137429	27.29089312	32.23398024	22.83160224	19.25780733	18.22072609	19.82765887	20.08502899
C18:1N9	Methyl oleate	ug/10^7	0.802615931	0.0236	65.84473225	58.87605272	67.70816133	41.95202685	89.57629504	77.99472275	74.15116518	75.21766733
C18:1TN9	Methyl elaidate	ug/10^7	1.073850595	0.789022504	1.744437216	1.659412764	2.153404918	0.928922198	1.781115316	0.938969298	1.07589988	2.244126786
C18:2N6	Methyl linoleate	ug/10^7	1.386723732	0.001777735	17.60531415	17.60272189	17.44946993	16.86357577	15.02270545	12.57761767	11.00004746	11.53296177
C18:2TTN6	Methyl linolelaidate	ug/10^7	1.108849737	0.2253	0.266276482	0.252992162	0.278369749	0.248725482	0.263038775	0.211262526	0.269630121	0.199716612
C18:3N3	Methyl linolenate	ug/10^7	0.856290018	0.141898458	1.824163608	1.881308214	1.925237398	1.675411579	2.602485602	2.177716347	1.828325151	1.923769958
C18:3N6	Methyl γ-linolenate	ug/10^7	1.150541644	0.2433	0.24329434	0.389082461	0.372586461	0.355569193	0.272958634	0.303483248	0.288018899	0.31805396
C20:0	Methyl arachidate	ug/10^7	1.195736205	0.023399477	1.489067995	1.323596026	1.680401804	1.476000141	1.283830949	1.144093948	1.281236532	1.28279745
C20:1N9	cis-11-Eicosenoic acid methyl ester	ug/10^7	1.057592983	0.7684	0.859629475	0.341172559	0.825233111	0.692895811	0.665137965	0.686734943	0.598057388	0.620936759
C20:2N6	cis-11,14-Eicosadienoic acid methyl ester	ug/10^7	1.614798321	0.000719433	0.4149408	0.475431767	0.495582527	0.420595677	0.33279746	0.272971371	0.250552655	0.26242551
C20:3N3	cis-11,14,17-Eicosatrienoic acid methyl ester	ug/10^7	1.190788002	0.42284895	0.306579953	0.435818742	0.431185322	0.321973286	0.2441545	0.256721435	0.259935994	0.495127248
C20:3N6	cis-8,11,14-Eicosatrienoic acid methyl ester	ug/10^7	1.738163282	0.000158656	2.512863687	2.782262099	2.777994863	2.549458649	1.847739777	1.532945946	1.353809076	1.376886916
C20:4N6	Methyl arachidonate	ug/10^7	1.782181721	0.000149099	10.88060049	11.84201661	11.50160977	10.73112755	7.753675315	6.35460423	5.408186084	5.708433665
C20:5N3	cis-5,8,11,14,17-Eicosapentaenoic acid methyl ester	ug/10^7	1.299963456	0.032923292	0.378015535	0.431393046	0.309091455	0.385222443	0.338894259	0.297323503	0.256884732	0.263639622
C21:0	Methyl heneicosanoate	ug/10^7	1.722731683	0.000110765	0.031421284	0.033732786	0.033267284	0.031951586	0.022683966	0.019431008	0.016042835	0.01752022
C22:0	Methyl behenate	ug/10^7	1.580133602	0.018101038	4.572316662	7.946062767	6.896606651	6.134837937	3.762170786	4.135535916	4.457536749	3.814163982
C22:1N9	Methyl erucate	ug/10^7	0.724299853	0.447159758	1.255313551	1.207738482	1.139865616	1.214875243	0.855670694	1.318171862	1.149592842	3.328219819
C22:2N6	cis-13,16-Docosadienoic acid methyl ester	ug/10^7	0.877001539	0.78835268	0.322903386	0.128985203	0.150528238	0.228500448	0.130501907	0.154686932	0.143728203	0.518535428
C22:4N6	Methyl docosatetraenoate	ug/10^7	1.57058204	0.0021	0.434447336	0.414767292	0.558657985	0.484866201	0.310367335	0.320656944	0.293939478	0.280155597
C22:5N3	Methyl docosapentaenoate	ug/10^7	1.842548541	0.011211644	2.911357195	4.634335548	3.940856692	3.541395995	2.6520548	2.348916394	1.932961667	1.222132011
C22:5N6	Methyl docosapentaenoate	ug/10^7	1.230419652	0.111991681	0.215414323	0.234866253	0.215665915	0.277051845	0.21147416	0.198923242	0.13672407	0.21928236
C22:6N3	cis-4,7,10,13,16,19-Docosahexaenoic acid methyl ester	ug/10^7	1.59958698	0.03504981	1.232426076	1.135647908	1.761959431	1.372500329	1.012606552	0.803842082	1.108076254	0.515446688
C23:0	Methyl tricosanoate	ug/10^7	1.269455694	0.108601698	0.044348826	0.039517452	0.057586979	0.035061114	0.034024251	0.032267249	0.036183234	0.036572553
C24:0	Methyl tetracosanoate	ug/10^7	1.476867882	0.003396908	0.746614293	0.862072667	0.97082324	0.739528749	0.546272389	0.527007455	0.569569933	0.604500165
C24:1N9	Methyl cis-15-tetracosenoate	ug/10^7	1.521253119	0.0239	4.062885448	5.327013523	4.993765685	4.541479295	3.471769165	3.582464246	4.248824101	3.727233834

Table S5. The sequence of LINC01606 promoter region and negative control region.

LINC01606 promoter sequences (2500 bp)

>hg38_DNA range=chr8:57216276-57218775 5'pad=0 3'pad=0 strand=+ repeatMasking=none

CTCTAGAGAGTCTGCTTTGAGACCACACGACCATCACCTCGCGGCTGCCTAACATTTACTGGTGGTGG
GGAACCCTCTCGGGCCCCGCTCTGCACGTGTGACGGAGAGGGACACCTGACAGGATTGAACGTGTGC
CAGAAGATCATCTGACGAGATGGAGTGTCTGGCGGAAGGAACATCTGACAGCCGAGTGTGACAGAGG
TAACATCTAAGCAGACTGCAGCGATTTCGGAAGGAACTGCTAAGAACATCTGGTGATCAGCATGAGGA
AACCTTAGGGTAGGGAGATTGCAGGGGGGGCTGGCAGGGTGAGGGAACCCGTGAGAAGACTGCAGG
ATGGGCAGTTGGACGCCCTAGGCAGATTGCAAGGCCTGCCCAAGAAACATCTGGGTGGAGGAAGTAT
TGGGCCCATGGTGCGCCGGACAAGACTGAGCGCTTGTCAGGGGAGGGAGCGGACACAACTAGCTGTT
TGGCAGAAGGAACACTCAGGTAGACTGCACGTTTAGCAGAAGGCCTAAGTGCGAAGATGGCGTGGTC
AACAGCAGAAAACCCTTAAGCAGGTCTCATGGTGGGCAGAAGGAACCTCTGAGCAGATTCCACGGTG
GGTAGACGGAACACCTAGGAAGATTGCATGCCGTGCAAAGGAAACAGCTGGAAAGAGGGAACATTT
GGGAGAGAAAACATCCGCTAAGATGGGGTGTTTGGCGGTAGGGACGTCCGCGGCGGTGGATCCATAG
AGGCCTGCACGCAGCAACCTGAGCTCTTGCCTCGTCAGAGGGAGTTTGACTGAGGGGCATGAGGCGG
AGGGAGAGAAACCGAGGCCACTTTAAGCGCGGGTCTCCCAGATGGTACCGGAGCAGGGAGGCGAGG
CAATAAGGTACACTTAGAAGAATGCCATGTGTGCAACTGGAGAGGTCAGGGGCATGCATTGTTCGACC
TTTGAGGAGAGGTCTCACACATTGGCAGGCTTCCAGGAGCCAGGGGTTGCGTCGCTTCCTCCCCTGA
GCCTTTTCACAGGGCGGGCCATCCACATGGGCAGTGGCCTGTCGGCACTTGGGAGGGACCGCAGGCG
CAGCGTGTTTCCTGGAGTCGTAGGGGTGCTCACTTGAGGCGTGCCTCCCTCGGCAGTTGGATGTTGCC
CAGGGAGGGTCATAGGACAGTGAAACCCGGCCGGTTTGCCTGTGAATGCGCAAGCGTGAACCCCCTC
GCCCACCCCCTGAGATCTCGGGGAGCTGCTCATCGCCACCTTCCAGGGTTTCTGTCTGTCTGGGGGAC
TGCCCGTCCCCGGCGGGGCTCCCAGCAATCATCTGTTATTTGAGAGAGGCAGTTTAATGGTGGCCTGA
CCCATCACCTCCTGGTCACCTGAGGTGTCAGGTGGGGTGTGGGCGTGGCCCTCTCCTGCGCTGCTCCT
GTCTGACCAGCCGCCCACGGTAACACAGAAACATCTGGCAGGATGGAGTGGTTGGCGGAAGACACAT
CTGACGAGATCGAATGTTTTGCAGAAGAGCCATGTGACCAGATCCCTGGTTTGGCAGAGGAGACATGC
GAGAAGACGGAACGTTTTGGCCGAGGAGACATCTGGCAGGGTGGAACGGAGGGAACAGGTGTGGCA
GAGAGAATATCTGAGAAGCTCGCAGGATTCCCAGAAGGAGCACTGGGAGTAGATTGCCTGTTTTCCCG
AAGGAACGGCAAGTTCCAAAGGTCAGAGGCTCATGTGAACTCTGTCGGGTTCAGGAGCAGCTCAGTG
TTGCTGGTGGCAAGAGTGTGGTACGAGAGTGGTTGGGCGTGGTTTGAATATCTAGAAAAGGCGAGCT
GAGGGTAGATTTTTGAACAGTCCGGAGTAGGAGGGGTAGAGACATATTCTATGGGCCACAGCCACATT
CTCAGGATGCTCGTGGCTGTAAATAGTAGATGACCCAAGTAAGAGCGGCTGAAGCCATAGGCACCAGA
ATTGTGTTGGCATCGTCGTGTCACGCTCACGTTTTCCCTTCCAGCTGTGCTGTGGGCAGTGTTTACTTC
ATGTCTGCTTTGCTGGCGGGCCAAGTAGCAACAGCTGCAGTCGGCGTGTTCTCTGAAGGCAGCGTGTG
AAGGGTGGAGGGGTTGCTCTTCTTCCCAGGTGTCTTTTGAAGAGGCAGTCAAGTCAGCAGCTCACAG
TGGCCTTCTGGTAACATCGTATGGGCTGGGTTACAGCACATGCCGACTGCTAAGCCAGTCACTGGGGA
AAACCATGTGATTGCTGTGATGAGCTTAGAATGATCATTCCTCCTTCAGGAGCTGGGGTGGGTTGAGG
GAACGATAAAGATCCTGATAACCTTGCATTTCTCCAGCGAGAAAGAAGAAATTCCGTGGATAGGGAGC
CAGCCAGGTTTCCTGCAGAGATTCACGGGAAGCTTTTGAGCAGGGGAGTCAGAAGATGAGCGGTGAG
TATTAGGACTTTCCAGTCACGGCGTGACGCAGGGTTTGGCCAGTTCTTCCTGTAAAGGGGCAGGTGGG
AAACGCT

LINC01606 promoter negative control sequences (2500 bp)

>hg38_DNA range=chr8:57230455-57232954 5'pad=0 3'pad=0 strand=+ repeatMasking=none

AGACTGACTTCCTCTTGAGGCTTATCACTAGCTCTGAATAGAAGCATTCCCAAATGCTGTCTAAGCAA
TTGTTGCGTCATGGGAATAAGTTGATAAATTCTCAGGGTTACATACTCCTTTAGATGCACTGGAACTT
TTCTCAGTATGTAGAATCAAGTGTAAGGAAAAAGGATTTGAAGGGGAAGGGGAGAAATAAAACAGG
ACTGAGCCTTACACGTGTTGACCAGAACAGATAATGTTTGGATTTTCTTTACTTTTTTCCCAGAGAAA
TCGAATAAAGAAGTTTGTTTCTGTTACCTAAAATCAACTCCCTCCCACCTTAAGTGGTTTTGTATAGA
GCCCATAGTTGAACTCTGTTTTCTACAGCAGAGATTCATTTTAATGTTTTTATATTATATCAAGGCAA
GTTTGATTTCTTTCTGGGTAACTATAGGCAGAATGTCAGCAATACTCCACGTATTAGCTATTAGAGAT
AAAACACTGTGAAACTCTAAAGGCCTTGATTATTGGGCCAATGAGATATTTATGCTGTGATCTGATG
GAGCAAAGCTTTGACAGGGTTATTGAGCCACCTTCAAAGTGTATCAGTGAAGTTCTTGGCTTGCTATT
TTAGATAAACAGCTTTCATTTTTACTAGTTGCTTGTACTTAATAAGGTTACTGGATTGATTCAAGTTGT
TTGCAATGCGTTGAAGGTGAAGAGTATTGTATCTAGTGTCCTCATAACACACTATTACTTTTCTGAGG
AAGGGTCTTGTCATTTTACCCTAGATATCAACAGAAAATATTCCATTTCTTATATATTAATTTTGCTCT
GCAATAAGGAAATATAACTACCAAAAATGCCATCGAAGGTTTTAGGTTGATGCTTATTTGTGTTTCCT
AATCTGTAGATAGCAGGGTCTTTTTTTAAATTTTTATTTTTTGTTTTGGTACAGGGTCGGCTTTGTTGC
CCACTTGGAGTGTAGTGGCACAACCTTGGCTCACTGCAACCTTCTCCTGAAGTACATATTAGCTCACC
TGGGCTCAAGTGATCCTCCCACCTCAGTCTTTTGAGTATCTGGGATTATTGGCATGTGCCACCAAGCC
TTGCTAATTTTTGTATTTTTTGTAGACACAGGATTTTGCCACGTTGGCCAGATTTGTCTTGAACTCCTA
AGCTCCAGTGATCCACCCACCTTGGCCTCCCAAAGTGCTAGGATTACACGTGTCACCATCGTGGCCA
ACCAGCAAGCTCTCTTTTAATGATTCAGAGTCTCAGGCCATAGCAGTATAAGTATCAAAACATCTATT
CCCAACTTGGAGTTGAGATTTCTAATACGAATTCTATTTTCTCCATAATCATAAAATAGCATCAGGAA
AAGTCTCTTGACATCTTAAAATACATATCACTGGAAAGTAGATCTTTTTGTCCTTCAGATAAACATAC
TGTAAAATTTGAAATGAAAATTATTCTTTGTACCACCATTCTGATTTTACCCAGGGTTAACACAAAAG
TTATAGCAGTACATGAAGCTCTGAGGCTTCAATGGGTGTAGATATAGACATCCAAACCTATAATTTT
ACATTAATCAGAATACTATTCATATAAATCACAAATGGCAAAACCATTTCTGAAGGTATTTGGTTTTA
TAGATTATAAAATTAAACTTCCTTATAATTGTGTCCAAACTGAAATATGCTTCCTGTACAAAAAAGCT
TTCTTCTAACAGTAAAGGCCTCCTCCCAACCCACCCGACACATACACACACACACACACACACACAC
ACACACACACACACACACGAAGAGAGAGAGAGAGAATTTAGAGCACTGATCTTTTCTCTCTTAGACTAA
TTATTTTAACATTATTTTCAGCCACTTTATTAATTGTGGGGTTAAGGGTAGGATAGATCTAATGCCCA
TTTCACATGTGTTGCAACATCAGAGATTATGGTTTTCATTAAAAACATCAGAGCTAAATTCCTTTTCA
AAAAAGACATTGTTACTGTTAGTACCTTGGGGAATGTTGTAGTCTTATGAATGCTGATACGTTTATTT
GAGCCAATTGAATATTATCTCTAATATAAACTATAATTTACTGTGTGATTCCTCTTCCTGTTTTACCTA
TATATACTCAAAGTGAATGATTGTGTTTAGTGAACATTTCTTTAGGTAATAAAAATTAATTTCATTTA
AGAAAGAAGTACAAAATAGTTATTAGAGCAAACTTTGGTATTTGTAATTTTGAAGTTACATACTTTTG
AAATAAACTTGGGCTTTCATGCCATGTTGGGTTTGAGGAAGATAGCAAATGTATAAATTGAGCTCTC
TAGTCATAACCTTGCTATATGTATTCCTGCTCATTAAAATACTTTGCGCCAGCAAAAATGATTTCCAA
CATATGTGTTTTGGATGTAATTAAGTAACTGTATAAAACTAAGTATGTTTTTTCTCCTTTCCCCAGTGA
CTGGAAAACTTTCATACTTTTAAGGTAATAATAAAATAATAATCTTTAAAGAGC
